# Comprehensive pan-cancer analysis of p62 reveals its contribution to shaping tumor microenvironment and anti-tumor immunity

**DOI:** 10.1007/s12672-025-04135-1

**Published:** 2025-11-25

**Authors:** Zahra Nayeri, Elahe Tavakol, Marveh Rahmati, Marco Cordani, Mojgan Djavaheri-Mergny, Vahid Shariati, Mohammad Amin Moosavi

**Affiliations:** 1 Department of Molecular Medicine, Institute of Medical Biotechnology, Engineering and Biotechnology, National Institute of Genetic, P.O. Box 14965/161, Tehran, Iran; 2 Shiraz University, Shiraz, Iran; 3 Cancer Biology Research Center, Cancer Institute, Tehran University of Medical Sciences, Tehran, Iran; 4 Department of Biochemistry and Molecular Biology, Faculty of Biological Sciences, Complutense University of Madrid, Madrid, Spain; 5 Instituto de Investigaciones Sanitarias San Carlos (IdISSC), Madrid, Spain; 6 Team “Metabolism, Cancer & Immunity”, Centre de Recherche des Cordeliers, INSERM UMRS1138, Sorbonne Université, Université de Paris, Paris, France; 7 Metabolomics and Cell Biology Platforms, Gustave Roussy Cancer Campus, Villejuif, France; 8 National Institute of Genetic Engineering and Biotechnology, P.O. Box 14965/161, Tehran, Iran

**Keywords:** p62/SQSTM1, Immune cell infiltration, Oncogenic pathways, Genomic instability, Tumor microenvironment, Pan-cancer

## Abstract

Sequestosome 1 (p62/SQSTM1) is a multifunctional adaptor protein whose dysregulation promotes tumorigenesis through autophagy, metabolic reprogramming, and immune modulation. However, its role across cancer types and impact on the tumor immune microenvironment remain poorly defined. Here, we performed a comprehensive pan-cancer analysis to delineate the molecular and immunological landscape of p62 across human malignancies. TCGA analysis revealed rare mutations and limited prognostic impact of genomic alterations but marked transcriptomic upregulation in LIHC, LUAD, BRCA, KIRP, KICH, KIRC, and READ, correlating with poor survival, advanced stage, and higher T classification, particularly in BRCA and LUAD. Pathway analysis showed strong positive associations between p62 and metabolic adaptation and stress tolerance pathways, including oxidative phosphorylation, reactive oxygen species, and DNA repair, in most cancers. Notably, high p62 expression inversely correlated with immune cell infiltration in most epithelial cancers, such as BRCA, COAD, ESCA, HNSC, KIRC, LIHC, LUAD, LUSC, PAAD, PRAD, READ, THCA, while coinciding with elevated expression of immunosuppressive checkpoints such as PD-L1, B7-H3, EBAG9, PVR, and TGFB1, supporting a link between p62-driven metabolic remodeling and an immune-excluded tumor microenvironment. In contrast, GBM, LGG, OV, SARC, and TGCT showed positive correlations between p62 and immunoscore, with enrichment of interferon and pro-inflammatory pathways, reflecting a distinct immune-activated phenotype. Collectively, these findings identify p62 as a central regulator of tumor metabolism and immunity, suggesting that its context-dependent activity may dictate the balance between immune suppression and activation across cancers, and highlight two natural-product-derived PB1 inhibitors (ZINC70669789 and ZINC08877690) as promising candidates for therapeutic development.

**Supplementary Information:**

The online version contains supplementary material available at 10.1007/s12672-025-04135-1.

## Introduction

Cancer progression is driven by a complex interplay between oncogenic signaling, metabolic adaptation, and immune regulation within the tumor microenvironment (TME) [24]. Among the key molecular adaptors coordinating these processes, *p62/SQSTM1* has emerged as a multifunctional signaling scaffold that integrates autophagy, oxidative stress, metabolic regulation, and inflammatory pathways [89, 101]. Despite extensive literature on its molecular roles, how these multifaceted functions of p62 integrate across distinct cancer contexts has remained largely unexplored.

Structurally, p62 contains several functional domains. The Phox and Bem1p (PB1) domain mediates homo- and hetero-dimerization as well as oligomerization, enabling the assembly of higher-order signaling complexes [45, 64, 91]. The ubiquitin-associated (UBA) domain recognizes polyubiquitinated substrates and plays a pivotal role in the ubiquitin–proteasome degradation pathway [36, 45]. The zinc finger (ZZ) domain binds N-terminal degrons [45, 47], targeting proteins for autophagic degradation. The Kelch-like ECH-associated protein 1 (KEAP1)-interacting region (KIR) disrupts the KEAP1–NRF2 complex, thereby activating the antioxidant response [32, 45], and the LC3-interacting region (LIR) which is crucial for selective autophagy [45, 72]. Collectively, these domains allow p62 to act as a central hub linking NF-κB, mTORC1, and NRF2 pathways, thereby affecting a wide range of cellular processes, including inflammation, metabolic and oxidative stress, genomic stability, proliferation, and cell death [27, 73, 89, 105].

Clinically, p62 overexpression has been identified as a potential prognostic marker in diverse cancers, such as leukemia [66], hepatocellular carcinoma [56], lung [86], pancreatic [87], ovarian [40], gastric [99], and colon cancers [48], and is often associated with poor outcomes. Furthermore, p62 expression can modulate responsiveness to radiotherapy, chemotherapy, and immunotherapy, highlighting its importance in cancer therapy and resistance mechanisms [7, 35, 70, 110].

Mechanistically, p62 coordinates multiple oncogenic and stress-response pathways. By activating NF-κB signaling, it promotes transcription of pro-survival and inflammatory genes; through mTORC1, it sustains anabolic metabolism and growth; and by stabilizing NRF2, it supports antioxidant defense and metabolic reprogramming [27, 45, 73, 89]. These interconnections position p62 as a critical mediator of tumor cell fitness under stress conditions.

However, under conditions where p62 facilitates the selective autophagic degradation of oncogenic proteins or damaged mitochondria, it may exert tumor-suppressive effects, as reported in hematological malignancies and early-stage hepatocellular carcinoma [8, 23, 66]. This context-dependent plasticity reveals a dual role for p62, functioning either as an oncogene or as a tumor suppressor depending on cancer type, disease stage, and metabolic context. Despite multiple mechanistic reports, there has been no systematic pan-cancer evaluation integrating these pathway interactions to delineate p62’s net effect across tumor types.

Beyond oncogenic signaling, growing evidence highlights a pivotal role for p62 in cancer immunity and TME remodeling. In breast cancer, p62 depletion enhances CD8⁺ and Th1 infiltration while reducing immunosuppressive macrophages and myeloid-derived suppressor cells [77]. In gastric cancer, p62 accumulation upregulates *PD-L1* expression via NF-κB signaling, thereby promoting immune evasion [93]. In hepatocellular carcinoma, elevated p62 expression is associated with increased infiltration of regulatory T cells (Tregs) and M2-polarized macrophages, alongside activation of inflammatory cytokine networks that support tumor progression [56]. Similarly, in lung adenocarcinoma, p62 overexpression suppresses immune cell infiltration and is linked to reduced responsiveness to immunotherapy [49]. Conversely, in colorectal cancer, p62 expression inversely correlates with FOXP3⁺ T cell density, although its overall impact on anti-tumor immunity remains unresolved [44].

Despite these insights, current knowledge remains fragmented and confined to specific cancer types, and the molecular connections between p62-mediated stress signaling, immunosuppression, and immune checkpoint regulation are not well elucidated. Additionally, emerging evidence suggests that p62 influences genomic stability, microsatellite instability (MSI), tumor mutational burden (TMB), and stem-like programs [60, 67, 98], which are critical hallmarks of tumor immunity and therapeutic responses [20, 42]. However, these relationships have not been comprehensively investigated.

To address these gaps, we conducted a comprehensive pan-cancer analysis integrating The Cancer Genome Atlas (TCGA) multi-omics and immune-deconvolution datasets to delineate the associations of p62 with cancer-promoting pathways, immune suppression, anti-tumor immunity, and genomic instability. In addition, to complement these systems-level findings, we performed in silico docking-based virtual screening of natural compounds targeting the PB1 domain, the structural interface responsible for p62 polymerization and signaling complex formation [81, 84]. This dual computational–structural framework provides both mechanistic and translational insights, establishing p62 as a pan-cancer biomarker and a potential therapeutic target.

## Materials and methods

### Data collection and gene/protein analysis of p62

The TCGA Pan-Cancer Atlas dataset (https://portal.gdc.cancer.gov/) was used in 2022 to extract data for 32 cancer types (Table S1). Only samples with complete information (clinical data, gene expression profiles and genomic alteration data) were selected, which include 8513 primary tumor samples from adrenocortical carcinoma (ACC), bladder urothelial carcinoma (BLCA), breast invasive carcinoma (BRCA), cervical squamous cell carcinoma and endocervical adenocarcinoma (CESC), cholangiocarcinoma (CHOL), colon adenocarcinoma (COAD), lymphoid neoplasm diffuse large b-cell lymphoma (DLBC), esophageal carcinoma (ESCA), glioblastoma multiform (GBM), head and neck squamous cell carcinoma (HNSC), kidney chromophobe (KICH), kidney renal clear cell carcinoma (KIRC), kidney renal papillary cell carcinoma (KIRP), brain lower grade glioma (LGG), liver hepatocellular carcinoma (LIHC), lung adenocarcinoma (LUAD), lung squamous cell carcinoma (LUSC), mesothelioma (MESO), ovarian serous cystadenocarcinoma (OV), pancreatic adenocarcinoma (PAAD), pheochromocytoma and paraganglioma (PCPG), prostate adenocarcinoma (PRAD), rectum adenocarcinoma (READ), sarcoma (SARC), skin cutaneous melanoma (SKCM), stomach adenocarcinoma (STAD), testicular germ cell tumors (TGCT), thyroid carcinoma (THCA), thymoma (THYM), uterine corpus endometrial carcinoma (UCEC), uterine carcinosarcoma (UCS) and uveal melanoma (UVM).

The “TCGAbiolinks” R package [11] was employed to download somatic mutation and RNA-sequencing gene expression profiling (HTSeq-count based). UCSC Xena Browser (https://xenabrowser.net/datapages/) was used to download modified clinical data from the Genomic Data Commons (GDC) TCGA cohorts. The rate of non-silent mutations of *p62* gene, including “Missense_Mutation,” “Frame_Shift_Del,” “Nonsense_Mutation,” “Splice_Site,” “Frame_Shift_Ins,” “In_Frame_Del,” “Translation_Start_Site, “In_Frame_Ins,” and “Nonstop_Mutation” in 32 cancers was determined using the “maftools” R package. The “lollipopPlot” was used to visualize different mutation types on the p62 protein structure. The percentage of *p62* mutations was calculated as the number of mutated samples divided by the number of filtered primary samples. CNVs, including deep deletions or amplifications, were analyzed using cBioPortal, which was generated by the GISTIC 2.0 algorithm and represented CNV as amplification (+ 2), gain or low-level amplification (+ 1), shallow deletions (− 1), and deep deletions (− 2) [6].

The “DESeq2” package was used to perform differential expression (DE) analysis in R version 4.1.3 (p ≤ 0.05 and absolute of fold change (|fold change|) > 1.2)). The “ggplot2” R package was applied to visualize difference in *p62* gene expression levels between tumor samples and their adjacent normal standard samples (log_2_ transformed and upper quantile normalized).

p62 protein and mRNA levels in various cells and tissues were examined using the genotype-tissue expression (GTEx) portal, function annotation of the mammalian genome (FANTOM), and human protein atlas (HPA) (https://www.proteinatlas.org/) databases. The UALCAN web tool (http://ualcan.path.uab.edu/analysis- prot.html) and the Clinical Proteomic Tumor Analysis Consortium (CPTAC) dataset were used for analyzing p62 protein levels between tumors and their normal samples.

### Association between *p62* gene expression and clinicopathological parameters

The association of *p62* gene expression levels with the main clinicopathological parameters, including age, gender and clinical T, N, and M stages, was analyzed by Kruskal–Wallis nonparametric analysis of variance (ANOVA) (among multiple groups) or Student’s *t*-test (between each pair of groups). In all cases, a *p* < 0.05 was considered statistically significant. Where applicable, p-values were adjusted using the Benjamini–Hochberg method to correct for multiple comparisons. To analyze the association of p62 expression levels with overall survival (OS), we first divided the samples into high-p62 and low-p62 groups based on the median gene expression level. Subsequently, the Cox proportional hazards model and log-rank test were used to calculate the hazard ratio (HR) using the survival R package. Furthermore, to explore the association of mutation, amplification, and deep deletion of *p62* with OS, cancers that represented each alteration in more than one sample were first selected, and then the HR was calculated among altered and non-altered samples. Survival curves were generated by GraphPad Prism software (v.9.2.0).

### Association between *p62* and hallmark signaling pathways

The hallmark pathways gene sets, which summarize key biological processes relevant to cancer, were collected from MSigDB v7.4 (http://www.gsea-msigdb.org/gsea/msigdb/index.jsp) [52]. The pathway activity scores were calculated for each sample using single-sample gene set enrichment analysis (ssGSEA), performed separately within each cancer type by the GSVA R package with default parameters (min.sz = 1, max.sz = Inf) [102]. Spearman’s correlations between *p62* level and pathway activity scores were calculated using the “correlation” R package (adjusted *p* < 0.05). To avoid sample size effects and reduce the dimensions of data, we applied T-distributed Stochastic Neighbor Embedding (t-SNE) by “Rtsne” and “ggplot2” R packages and visualized the clustering cancers across a dimensional space. Hierarchical and k-means clustering were based on the complete linkage method with Euclidean distance. Correlation heatmaps were generated with the “Complexheatmap” R package (https://jokergoo.github.io/ComplexHeatmap-reference/).

### Correlation between *p62* expression and genomic instability

The genome instability scores, including TMB, the total number of mutations per megabyte of each tumor sample, and MSI, an indicator of repetitive sequences of mono- and oligonucleotides, were examined using Spearman’s rank correlation coefficient, with statistical significance set at *p* < 0.05, and then the “circlize” R package was used to visualize circular correlation heatmaps.

### Correlation between *p62* expression and anti-tumor immunity

Two approaches were used to evaluate the correlation between *p62* expression and tumor immunity. First, the rate of immune/stromal cells infiltration and tumor purity were calculated based on the gene expression data using the “ESTIMATE” R package. All correlations were adjusted for tumor purity where applicable, following established approaches in immune deconvolution analyses [104]. Then, the tumor immune estimation resource (TIMER, http://timer.cistrome.org/), which quantifies the relative levels of different cell types based on their gene expression patterns, was used to estimate the fraction of different immune cells in the TME of TCGA samples. The “correlation” R package applied to calculate Spearman correlation coefficients in all cases, with statistical significance set at *p* < 0.05.

### Docking-based virtual screening

We conducted a docking-based virtual screening to identify potential inhibitors targeting p62 to enhance the anti-tumor immunity response. The crystal structure of p62 was obtained from RCSB PDB (PDB ID: 4MJS). In addition, to confirm our findings and account for missing residues in 4MJS, a full-length modeled structure of p62 was generated using SWISS-MODEL (https://swissmodel.expasy.org/).

Molecular structures of natural products were retrieved from the ZINC database and energy-minimized using Open Babel (https://github.com/openbabel/openbabel/releases). Docking simulations for all ligands were performed using AutoDock Vina 1.1.2 package (https://github.com/ccsb-scripps/AutoDock-Vina). The docking grid box for the 4MJS crystal structure was maximized to fully encompass the PB1 domain (center_x = 25.5005, center_y = 5.8695, center_z = 12.5749; size_x = 26.1832407475, size_y = 37.3697505188, size_z = 30.8049596095). For the SWISS-MODEL structure, the grid box was defined based on the sequence alignment corresponding to the PB1 domain (center_x = –8.268, center_y = 16.678, center_z = 11.896; size_x = 41.25, size_y = 47.25, size_z = 34.5).

Additionally, the docking protocol was validated prior to large-scale virtual screening. Because our target structure lacks a co-crystallized ligand, we performed a control redocking using the well-characterized CDK2–staurosporine complex (PDB ID: 1AQ1), which has been previously used for AutoDock Vina validation [39]. The native ligand was re-docked into its receptor, and the root-mean-square deviation (RMSD) between the experimental and predicted poses was calculated using DockRMSD (https://zhanggroup.org/DockRMSD/). Besides, in the absence of site-specific reference ligands for our protein, we analyzed the distribution of docking affinities across the screened library and selected compounds exhibiting the most favorable binding energies as top candidates for further evaluation, following the general guidance for establishing pragmatic cut-offs in large-scale docking campaigns [4, 58].

SwissTargetPrediction (http://www.swisstargetprediction.ch/) and ADVERPred (https://www.way2drug.com/adverpred/) were used to evaluate the potential off-target and side effects of the selected ligands. Targets with a SwissTargetPrediction probability score greater than 0.2 were considered potential targets for each ligand. This threshold was chosen to ensure a higher likelihood of biologically relevant interactions while minimizing false-positive predictions. Ligands lacking high-probability predicted targets and exhibiting minimal side effects were selected for further analysis. In ADVERPred, Pa (probability to be active) and Pi (probability to be inactive) were interpreted according to the server guidelines, and the same conservative probability threshold was applied in SwissTargetPrediction to ensure consistency across both analyses. The final selection of ligands was assessed for optimized pharmacokinetic profiles, determined by evaluating the minimal likelihood of violating established drug-likeness parameters, including the Lipinski, Ghose, Veber, Egan, and Muegge rules, using SwissADME (http://www.swissadme.ch/), using SMILES structure of selected ligands. Finally, visual illustrations of the interactions between the selected ligands and p62 were generated using Discovery Studio Visualizer 19.1.0.219.

## Results

### Low frequency of *p62* genomic alterations with no prognosis value were detected across TCGA samples

**Fig. 1:**
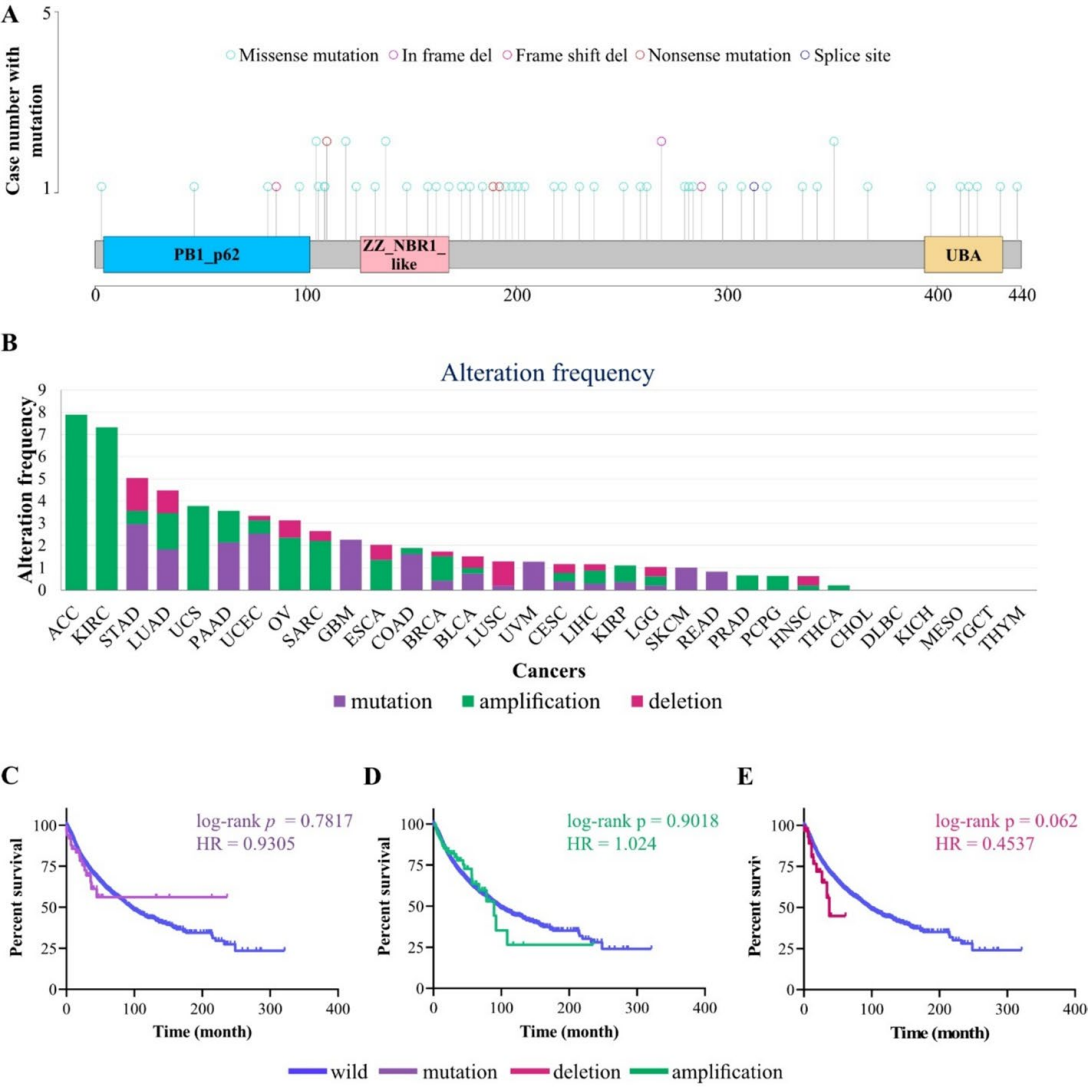
Genomic alteration landscape of *p62*. **A** The lollipop Plot displays mutation sites and case numbers of p62 across pan-cancer samples. Missense mutations have the highest frequency and are the major types of mutations. **B** The bar plot represents the frequency of mutations and CNV for p62 in different types of cancer. Kaplan–Meier plots displaying associations between overall survival (OS) and mutation status (**C**), amplification status (**D**), or deletion status (**E**) of p62 across pan-cancer samples

Mutation analysis (Fig. 1A and S1) revealed that the median rate of *p62* somatic mutations among TCGA samples was 0.69%, which mostly included missense mutations, a pattern consistent with the general mutational landscape observed across frequently mutated genes in pan-cancer cohorts (1). These mutations were distributed in the PB1 (aa. 21-103), the ZZ (aa. 128-163), the UBA (aa. 386-440), and LIR (aa. 321-345) domains of p62 protein (Fig. 1A and S2). STAD (2.96%), UCEC (2.53%), GBM (2.26%) and PAAD (2.13%) are top cancers with higher rate of *p62* mutation (Fig. 1B). No mutations were detected in TCGA samples of 16 cancers, including ACC, CHOL, DLBC, ESCA, HNSC, KICH, KIRC, MESO, OV, PCPG, PRAD, SARC, TGCT, THCA, THYM, and UCS (Fig. 1B). In addition, 1.34% of the TCGA samples contained CNV (deep deletion and amplification) for *p62*. ACC (7.89%), KIRC (7.32%), and UCS (3.77%) were cancers with a high amplification rate (Fig. 1B), whereas STAD (1.48%), LUSC (1.09%), and LUAD (1.02%) are top-3 cancers with a higher frequency of deletion (Fig. 1B). No CNV was observed in TCGA samples from 10 cancers, including CHOL, DLBC, GBM, KICH, MESO, READ, SKCM, TGCT, THYM, and UVM (Fig. 1B). Notably, Kaplan–Meier analysis revealed that neither *p62* mutations nor CNV were associated with OS among TCGA cancer patient samples (Fig. 1C–E), suggesting that genomic alterations have no significant prognostic value across cancers (Fig. 1C–E).

### The heterogeneous expression pattern of *p62* is associated with clinicopathological factors in patients with cancer

**Fig. 2:**
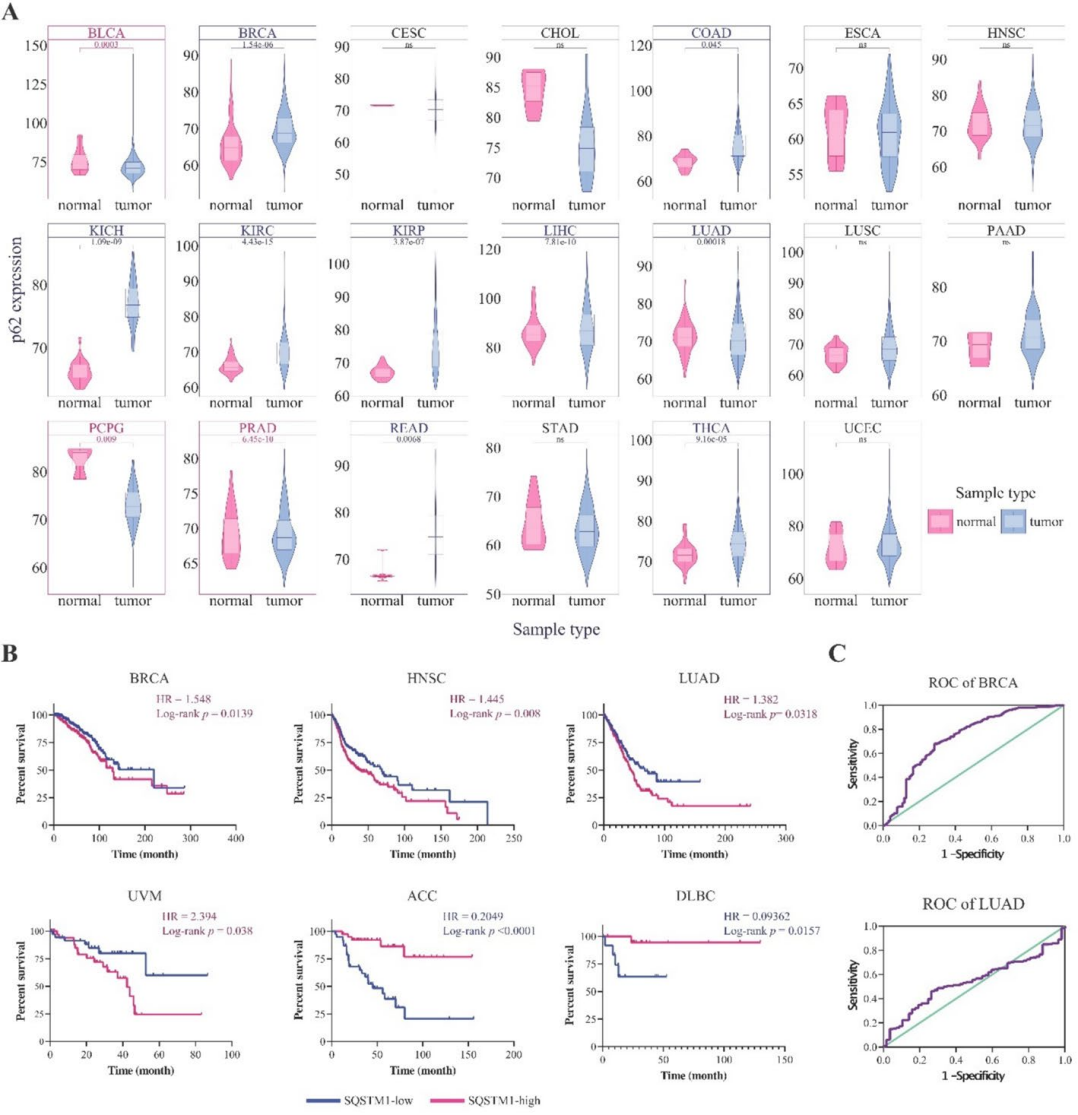
Expression levels of *p62* across human TCGA cancers. **A** Violin plot representing the expression level of *p62* in primary tumors versus adjacent normal samples of TCGA cancers. The blue boxes display cancers in which *p62* expression increased in primary tumor samples and pink boxes represent cancers in which *p62* expression increased in adjusted samples. **B** Association between *p62* expression and overall survival (OS) in patients with different cancer types. GraphPad software was used to conduct overall survival analyses. The *p* < 0.05 was considered a significant level and only the survival map and Kaplan–Meier plots with significant results are shown. **C** ROC curves of *p62* expression in tumor samples from BRCA and LUAD patients compared to normal samples

To obtain a comprehensive view of p62 gene/protein expression patterns in normal tissues, we examined GTEx expression (Fig. S3A), FANTOM5 (Fig. S3B), and HPA databases (Fig S3C). p62 is ubiquitously expressed across all normal tissues (with higher levels in the skeletal muscle, adrenal gland, and liver) and is distributed in subcellular compartments such as lysosomes, cytosol, endosomes, endoplasmic reticulum (ER), mitochondria, cell surface, and extracellular space (Fig. S2 and S3), reflecting its diverse biological functions in the human cells. Differential expression analysis between the tumor samples and their adjacent normal samples showed that *p62* mRNA was upregulated in LIHC, KIRP, KICH, KIRC, READ, LUAD, and BRCA tumors (Fig. 2A), whereas it was downregulated in BLCA, PRAD, and PCPG (Fig. 2A). A high protein level of p62 was also observed in cancers, including breast and lung cancers (Fig. S4), validating the gene expression results (Fig. 2A).

To determine the clinical importance of *p62* transcriptional changes, we analyzed OS in high and low *p62* expression groups for each cancer type (see Materials and Methods Sect. 2.2). The results demonstrated that high *p62* levels correlated with shorter OS (poor prognosis) in BRCA (HR = 1.548, *p* = 0.0139), HNSC (HR = 1.445, *p* = 0.008), LUAD (HR = 1.382, *p* = 0.0318), and UVM (HR = 2.394, *p* = 0.038). In contrast, longer OS (good prognosis) was observed in the high-p62 ACC (HR = 0.2049, *p* < 0.0001) and DLBC (HR = 0.09362, *p* = 0.0157) groups (Fig. 2B). In addition, ROC curve analysis revealed that high levels of *p62* can be considered a diagnostic factor in BRCA and LUAD (Fig. 2C).

**Fig. 3:**
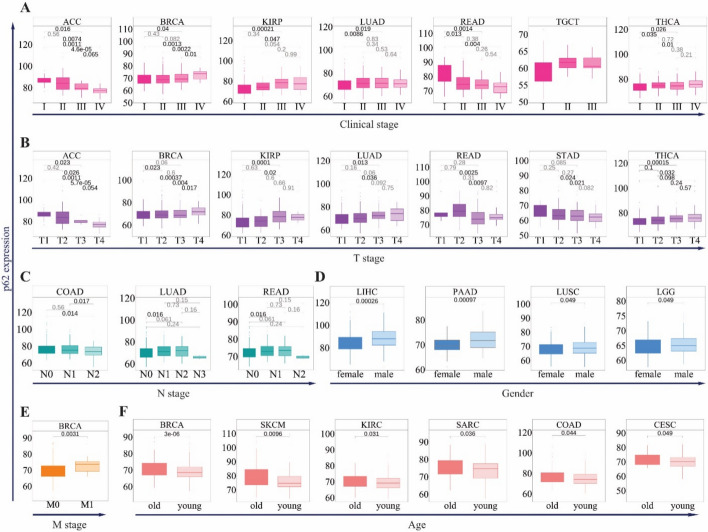
Relationship between *p62* gene expression and clinicopathological parameters. Association of *p62* expression with: **A** main pathological stages (stage I, stage II, stage III, and stage IV), **B** T stages (T1, T2, T3, and T4), **C** N stage (N0, N1, N2, and N3); **D** sex (male and female); E) M stage (M0 and M1); and F) age (old (< 65 years) and young (> 65 years)). For each parameter, only cancers with significant differences (*p* < 0.05) are shown. The ggplot2 package was used to illustrate the expression levels of samples based on the normalized HTSeq-count (log_2_ transformed and the upper quantile normalized)

*p62* expression was also significantly associated with clinical stage in seven cancers, including ACC, BRCA, KIRP, LUAD, READ, THCA, and TGCT (Fig. 3A). In BRCA, KIRP, LUAD, THCA, and UVM, high expression of *p62* was associated with advanced stages of tumorigenesis (stages III and IV) (Fig. 3A), whereas in ACC, TGCT, and READ, low expression was correlated with advanced stages (Fig. 3A). These trends were consistent with T stage data, where p62 expression was elevated in late-stage tumors (T3–T4) for BRCA, KIRP, LUAD, and THCA, but decreased in early-stage ACC and READ (T1–T2) (Fig. 3B). In the M stage, *p62* showed a significant association only with BRCA, which was higher in M1 samples (Fig. 3E). In contrast, three cancers, LUAD, COAD, and READ, are associated with the N stage (Fig. 3D). Furthermore, *p62* gene expression showed a gender- and age-specific pattern; its high level was associated with male samples of LIHC, PAAD, LUSC, and LGG (Fig. 3E), and was higher in the ≥ 65 years age group than in the < 65 years group in BRCA, SKCM, KIRC, SARC, COAD, and CESC (Fig. 3F).

### *p62* is linked to oncogenic pathways, genomic instability, and cancer stemness

**Fig. 4:**
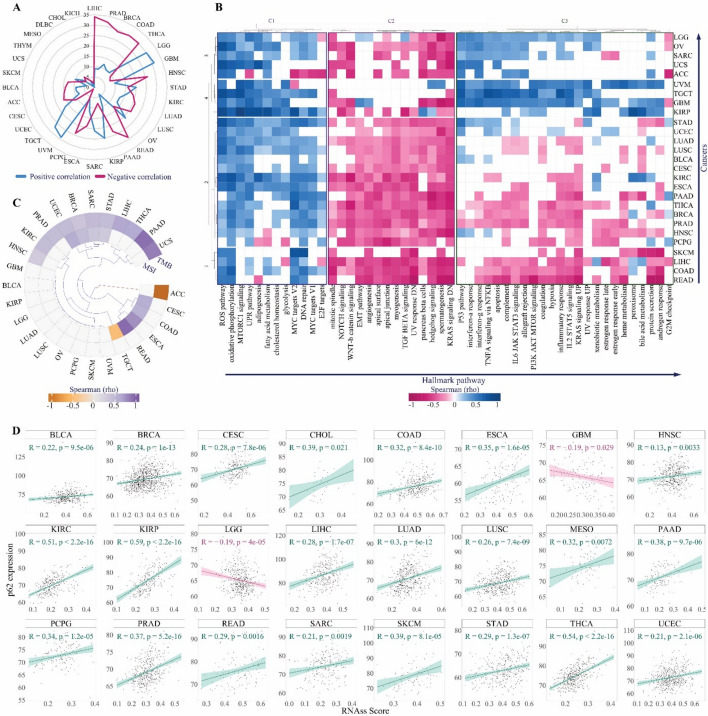
Functional effects of *p62* on oncogenic pathways, genomic instability, and cancer stemness. **A** Radar plot for activation (positive correlation, blue) or inhibition (negative correlation, pink) of oncogenic hallmark pathways correlated with *p62* across all 32 TCGA cancer types. **B** Heatmap of the Spearman correlation coefficient between *p62* and hallmark pathways among the filtered cancers. The k-means clustering in the columns demonstrates the key activation or inhibition pathways associated with *p62*. The k-means clustering on rows indicates the key cancers enriched for the activation or inhibition of *p62*. **C** Circular heatmap representing the Spearman correlation coefficient between p62 and genomic instability parameters (TMB and MSI scores). The purple spectrum represents positive correlations, whereas the brown spectrum represents negative correlations. **D** Scatter plot showing the correlation between the RNAss (stemness) score and p62 gene expression level. The Green and pink lines represent positive and negative correlations, respectively

Pathway analysis using MSigDB hallmark gene sets revealed that *p62* expression has tight crosstalk with the main oncogenic processes in LIHC (43 pathways), PRAD (41 pathways), BRCA (41 pathways), COAD (40 pathways), and THCA (40 pathways) (Fig. 4A and Table. S2). In contrast, p*62* expression displayed the least association with the hallmark pathways in MESO (7 pathways), DLBC (7 pathways), CHOL (4 pathways), and KICH (3 pathways) (Fig. 4A and Table. S2). To avoid sample size effects, cancers with the lowest number of samples and less significant correlations (i.e., CHOL, KICH, DLBC, MESO, and THYM) were filtered out using the t-SNE classification (Fig. S5). For the remaining 27 cancers, a clustered heatmap of hallmark pathway correlations revealed three clusters of pathways, two of which revealed a specific correlation pattern (Fig. 4B): cluster 1 that involves mostly positive correlations, which involved oxidative phosphorylation (26 cancers), reactive oxygen species (ROS) pathway (25 cancers), unfolded protein response (UPR) (24 cancers), mTORC1 signaling (in 21 cancers), MYC targets V2 (22 cancers) and MYC targets V1 (18 cancers), DNA repair (21 cancers), fatty acid metabolism (17 cancers), cholesterol homeostasis (16 cancers), adipogenesis (14 cancers), glycolysis (13 cancers) and E2F targets (10 cancers) pathways; and cluster 2 that shows negatively correlated pathways (cluster 2) that consist of KRAS signaling DN (25 cancers), spermatogenesis (24 cancers), hedgehog signaling (22 cancers), TGFβ signaling (22 cancers), the apical junction (21 cancers), UV response DN (21 cancers),, myogenesis (20 cancers), pancreas beta cells (20 cancers), angiogenesis (18 cancers), Wnt/β-catenin signaling (20 cancers), apical surface (19 cancers), mitotic spindle (19 cancers), angiogenesis (18 cancers), epithelial-mesenchymal transition (EMT) (17 cancers) and NOTCH signaling (14 cancers) (Fig. 4B and Table. S2). Therefore, *p62* represents both positive and negative crosstalk with hallmark cancer pathways, underling its dual oncogenic and tumor-suppressive roles.

Given the distinct association of p62 with mitotic spindle and DNA repair activities, both crucial for maintaining genome integrity [61, 75], we further analyzed the its correlation with genome instability signature factors, TMB and MSI. Our analysis revealed that p62 expression positively correlates with TMB in 11 cancer types, including HNSC, BRCA, PRAD, KIRC, STAD, UCEC, SARC, THCA, LIHC, UCS, and PAAD, while showing a negative correlation in ACC. (Fig. 4C). Additionally, p62 expression positively correlates with MSI in eight cancers, such as READ, ESCA, UCEC, CESC, COAD, BRCA, SARC, and STAD, but negatively in TGCT (Fig. 4C). Notably, in BRCA, SARC, STAD, and UCEC, *p62* expression was linked to both TMB and MSI (Fig. 4C). Moreover, *p62* expression shows a positive associated with RNA-based stemness scores (RNAss) in 22 cancers and with DNA methylation-based stemness scores (DNAss) in 4 cancers, while exhibiting a negative correlation with both RNAss and DNAss in LGG (Fig. 4D, S6, and Table. S3). Given that genomic instability and stemness can significantly influence tumor-immune interactions, we next examined how p62 expression relates to the immune landscape across cancers.

### *p62* expression level is negatively correlated with anti-tumor immunity factors

**Fig. 5:**
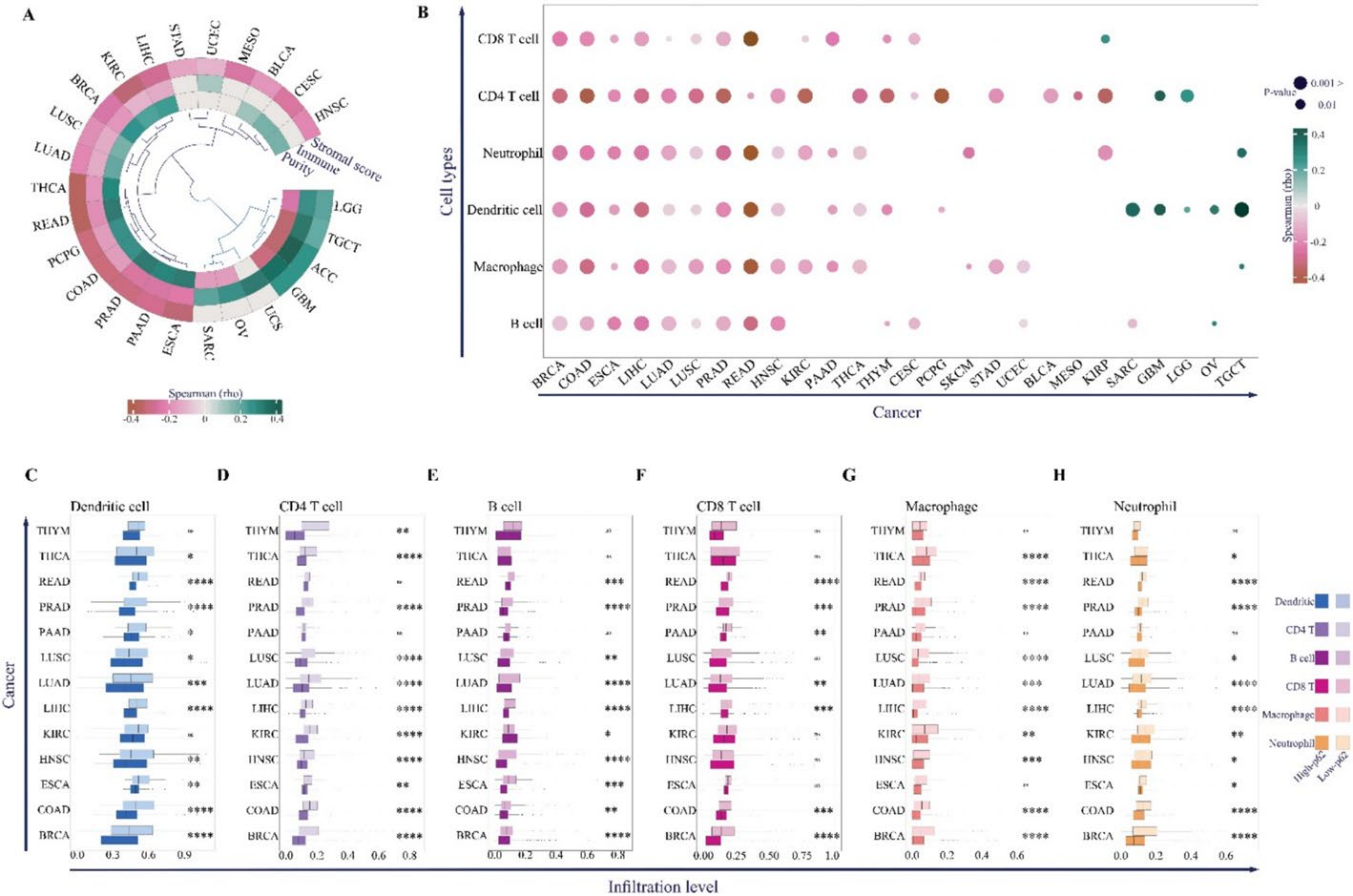
Association between *p62* expression and anti-tumor immunity determinants. **A** Circular heatmap representing the Spearman correlation coefficient between *p62* expression and TME-related scores. **B** The bubble plot represents the Spearman’s correlation coefficient between p62 and immune cell infiltration. The color represents the correlation coefficients, and the size of each bubble represents the FDR *p*. FDR *p* < 0.05 was considered a significant level, and only the correlation coefficient with FDR *p* < 0.05. Circlize and ggplot2 R packages were used to plot correlation results. The box plots represent the infiltration levels of dendritic cells (**C**), CD4 + T cells (**D**), B cells (**E**), CD8 + T cells (**F**), macrophages (**G**), and neutrophils (**H**) in high-p62 and low-p62 samples of TCGA cancers. Colors represent different immune cell types, and transparency represent high-p62 and low-p62 groups. * p 0.05, ** *p* < 0.01, *** *p* < 0.001, **** *p* < 0.0001. ns indicates no significant difference

The TME consists of immune cells and fibroblasts, which together shape tumor immunity and the therapeutic response of cancer cells [80]. We found that *p62* expression negatively correlated with stromal and immune scores in most cancers, including LUSC, LUAD, KIRC, LIHC, BRCA, PCPG, COAD, THCA, READ, ESCA, PAAD, and PRAD (Fig. 5A). Conversely, the tumor purity score, calculated from ESTIMATE [104], was positively correlated with these cancers, leading us to hypothesize that *p62* overexpression may suppress immune cell infiltration (Fig. 5A). However, these associations may be influenced by confounding factors such as tumor purity, cancer type heterogeneity, or genomic instability metrics like TMB and MSI.

Thus, we analyzed the cell infiltration of six major immune cell groups: B cells, CD4 + T cells, CD8 + T cells, macrophages, neutrophils, and dendritic cells (Fig. 5B). Similar to the results of the immune score (Fig. 5A), *p62* expression levels were negatively correlated with all six immune cell types in BRCA, COAD, ESCA, LIHC, LUAD, LUSC, PRAD, and READ (Fig. 5B). HNSC, KIRC, PAAD, THCA, and THYM were also negatively correlated with more than half of the immune cells (Fig. 5B).

Notably, infiltration levels of dendritic cells and antigen-presenting cells involved in the activation of cytolytic T cells, CD8 + T cells, B cells, macrophages, and neutrophils were higher in the low expression group than in the high expression group (Fig. 5C–G). These results confirm that high p62 levels may act as a blocker of the cell-mediated anti-tumor immunity response. To further understand the mechanisms through which p62 may contribute to immune evasion, we analyzed its correlation with immunosuppressive checkpoint genes.

**Fig. 6:**
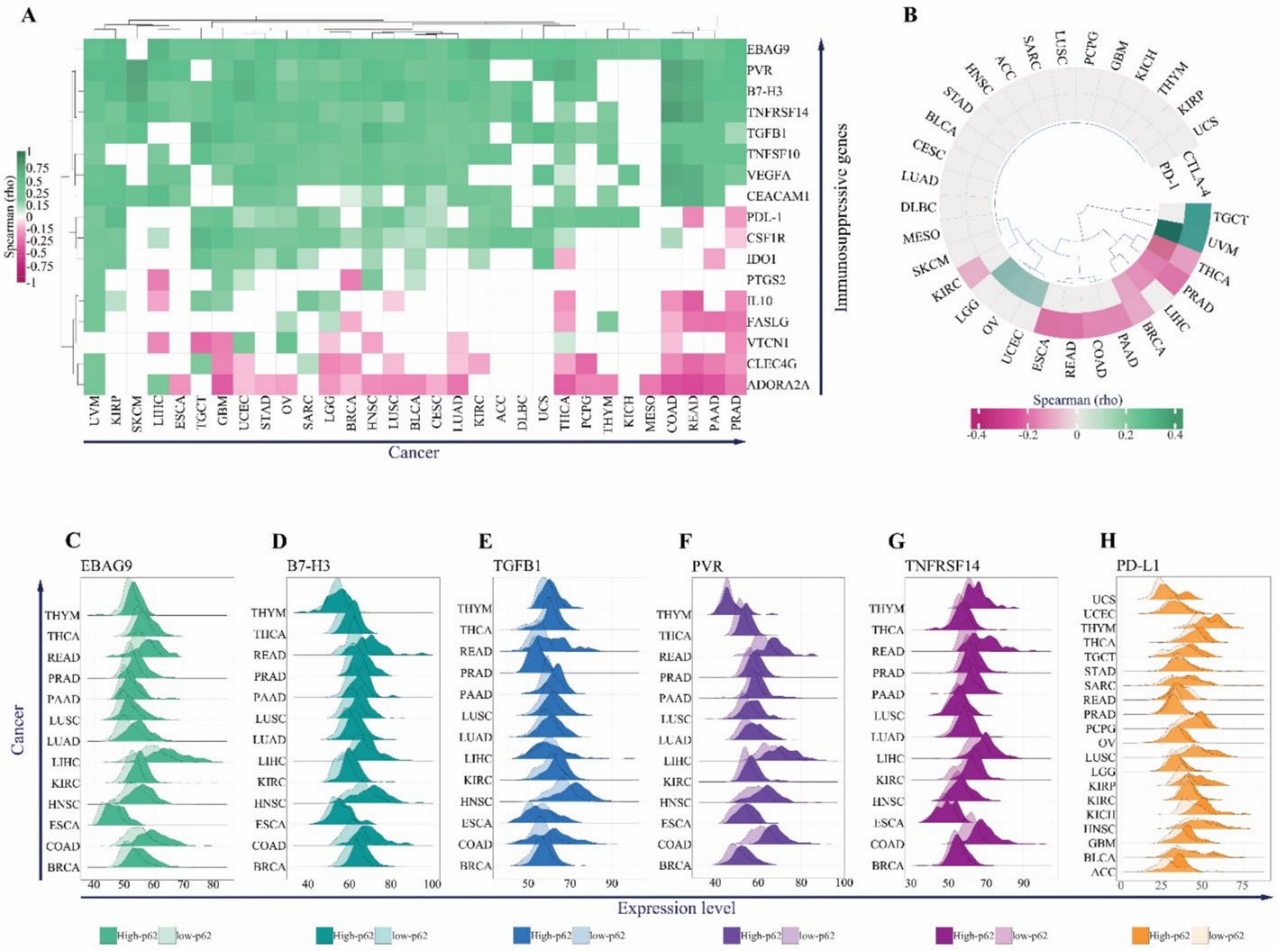
Association between *p62* and immunosuppressive gene expression. Heatmap of Spearman’s correlation coefficient between *p62* and immunosuppression genes (**A**) or chemokine receptor genes CTL-4 and PD-1 (**B**). For each pair of correlations, only the correlation coefficient with FDR *p* < 0.05 has been illustrated. The density plots for expression levels of *EBAG9* (**C**), *B7-H3* (**D**), *TGFB1* (**E**), *PVR* (**F**), *TNFRSF14* (**G**), and *PD-L1* (**H**) between high-p62 and low-p62 cancers groups Colors represent different immunosuppressive genes and transparency represents high-p62 and low-p62 groups

On the other hand, overexpression of p62 may limit anti-tumor immune responses via overexpression of immunosuppressive checkpoints. To examine this possibility, we collected a confirmed list of immunosuppressive genes and found a positive correlation between *p62* and the main suppressors of T-cell immune function on the tumor cell surface, including *EBAG9*, *PVR*, *B7*-H3, *TNFRSF14*, *TGFB1*, and *PD-L1*, in most cancers (Fig. 6A).

However, a negative correlation was observed between p62 and two well-known immunotherapy targets, T-lymphocyte-associated antigen 4 (*CTLA-4*) and programmed death 1 (*PD-1*) (Fig. 6B). The negative correlation between p62 and these two cytolytic T cell blockers might be related to the decreased level of immune cell infiltration observed in our analyses (Fig. 5B). Finally, we confirmed that the expression levels of the top immunosuppressive genes, *EBAG9*, *PVR*, *TGFB1*, *B7-H3*, *TNFRSF14,* and *PD-L1*, were significantly higher in the high-p62 group than in the low-p62 group in most cancers (Fig. 6C–H).

### Enhancing immunotherapy: targeting p62 with high-affinity natural product inhibitors

Our results suggest the potential therapeutic advantage of targeting p62 to improve the effectiveness of immune checkpoint inhibitors (ICIs) therapy and immunotherapy. Besides, p62's positive correlation with key oncogenic pathways prompted us to identify novel inhibitors that can mitigate its activity.

First, validation of the docking protocol was performed using the CDK2-staurosporine complex (PDB ID: 1AQ1). The redocking experiment showed binding affinity of – 12.2 kcal/mol, with RMSD = 0.422 (Fig. S7 and Table. S4), confirming the reliability of our computational pipeline.

**Fig. 7:**
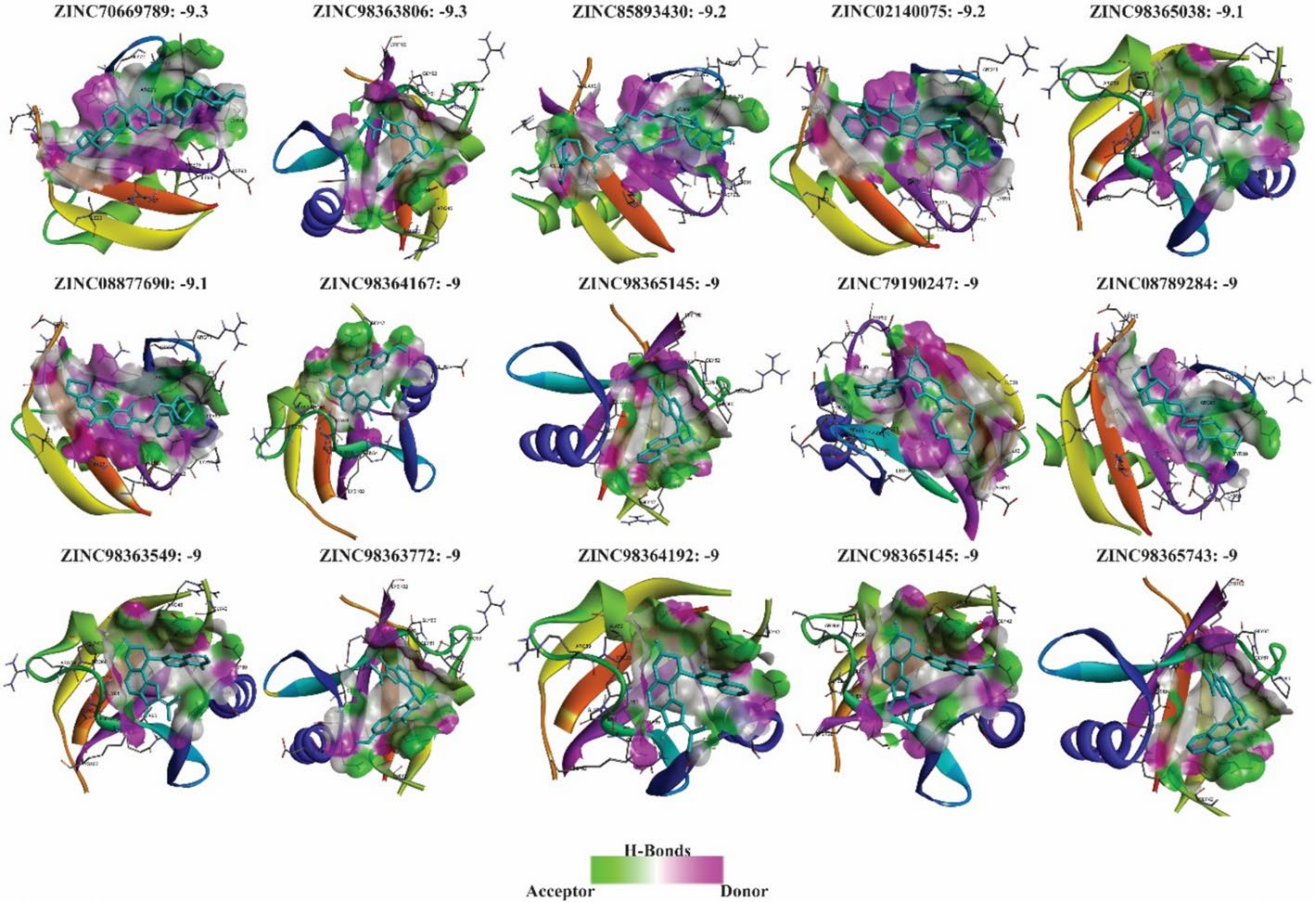
Identification of potential p62 inhibitors through virtual screening. Docking-based virtual screening of natural products targeting the PB1 domain of p62 identified 15 compounds with high binding affinity (≤ – 9 kcal/mol)

A large-scale virtual screening of natural compounds from the ZINC database was then carried out against the PB1 domain of 4MJS. Initial screening identified multiple ligands with binding energies ranging from – 3 to – 9.3 kcal/mol. Molecules with the strongest affinities (≤ –9 kcal/mol) were shortlisted, resulting in 15 top candidates, none of which exhibited high off-target probabilities (> 0.2), suggesting minimal nonspecific interactions (Fig. 7; Tables. S5 and S6). We validated these results by docking against the PB1 domain of p62 using the SWISS-MODEL structure, which all showed strong binding (Table. S7).

To further refine our selection, an ADVERPred analysis was performed to assess the potential toxicity of compounds, leading to the identification of four molecules—ZINC70669789, ZINC85893430, ZINC08877690, and ZINC79190247—with no predicted adverse effects, including hepatotoxicity, cardiac failure, myocardial infarction, or arrhythmia. Conversely, other compounds (ZINC02140075, ZINC08789284, ZINC217598959, ZINC217623094, ZINC217626420, ZINC217664974, ZINC217667805, ZINC217757795, ZINC217768522, ZINC217822045, and ZINC217830196) exhibited varying degrees of predicted side effects and were consequently removed from our list (Table 1).

**Table 1:** Toxicity prediction results from ADVERPred analysis

Zinc ID	Pa	Pi	Side effect
ZINC02140075	0.454	0.222	Hepatotoxicity
	0.335	0.14	Cardiac failure
	0.324	0.155	Myocardial infarction
ZINC08789284	0.417	0.246	Hepatotoxicity
ZINC217598959	0.828	0.053	Hepatotoxicity
ZINC217623094	0.732	0.088	Hepatotoxicity
	0.325	0.264	Arrhythmia
ZINC217626420	0.662	0.115	Hepatotoxicity
ZINC217664974	0.457	0.22	Hepatotoxicity
	0.309	0.291	Arrhythmia
ZINC217667805	0.803	0.059	Hepatotoxicity
ZINC217757795	0.738	0.085	Hepatotoxicity
	0.383	0.2	Arrhythmia
ZINC217768522	0.5	0.195	Hepatotoxicity
	0.325	0.265	Arrhythmia
ZINC217822045	0.349	0.301	Hepatotoxicity
	0.328	0.259	Arrhythmia
ZINC217830196	0.574	0.158	Hepatotoxicity
ZINC85893430	_		
ZINC79190247	_		
ZINC70669789	_		
ZINC08877690	_		

**Fig. 8.**
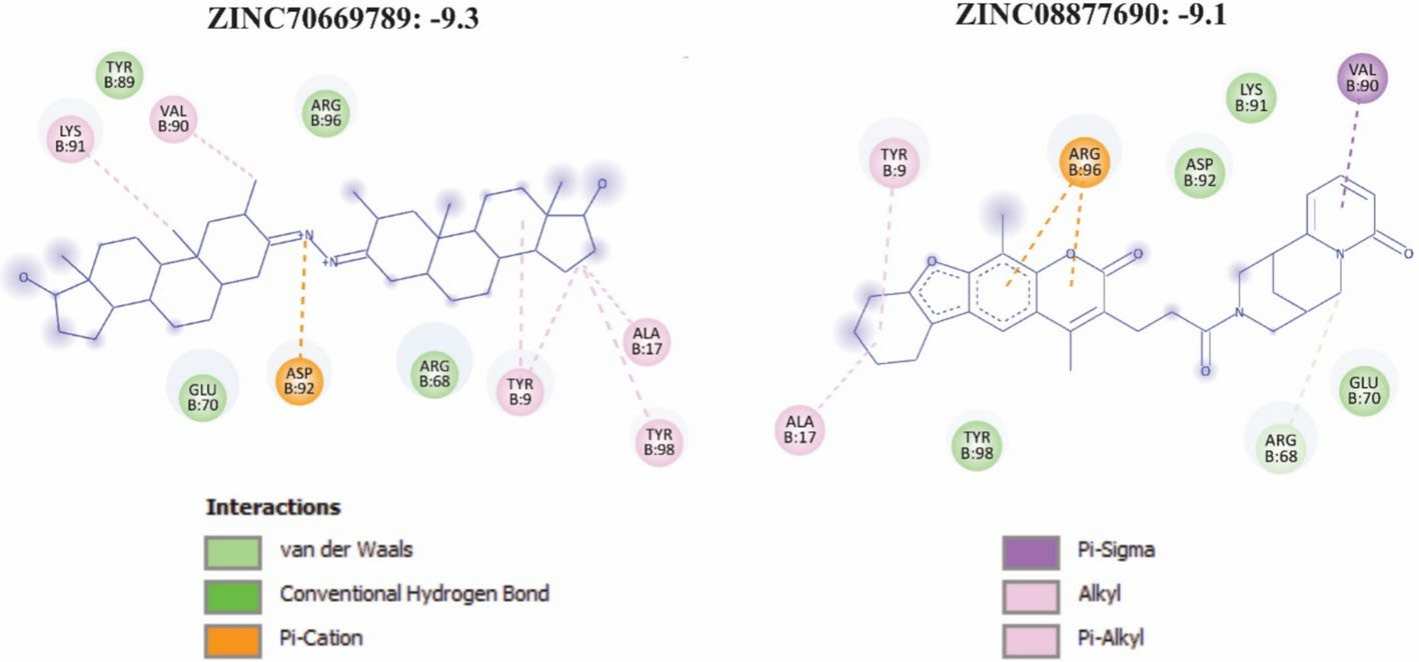
2D interaction diagrams of selected natural product inhibitors targeting the PB1 domain of p62. ZINC70669789 and ZINC08877690 demonstrate strong binding affinities of – 9.3 and – 9.1 kcal/mol, respectively. The diagrams illustrate key molecular interactions within the PB1 domain, including hydrogen bonds, Pi-Cation interactions, and hydrophobic contacts such as alkyl and Pi-alkyl interactions

Subsequently, we selected the molecules with optimized pharmacokinetic profiles. This was determined by assessing the minimal likelihood of violating established drug-likeness parameters, including Lipinski, Ghose, Veber, Egan, and Muegge rules (Table 2 and S5). Based on this, the two compounds ZINC85893430 and ZINC79190247, which had the highest deviations from the Ghose violations and Muegge violations, were removed. As a result, two compounds, ZINC70669789 with affinity scores of -9.3, and ZINC08877690 with affinity scores of -9.1, were selected as potential final inhibitors of P62. (Fig. 8, Table 2, and S5).

**Table 2:** Deviation results from drug similarity parameters based on SwissADME analysis

Zinc ID	Lipinski violations	Ghose violations	Veber violations	Egan violations	Muegge violations
ZINC70669789	1	2	0	0	0
ZINC85893430	2	3	1	1	2
ZINC08877690	1	2	0	0	0
ZINC79190247	1	3	0	0	1

Interaction analysis revealed that ZINC70669789 (– 9.3 kcal/mol) forms a key π–cation interaction with Asp92, accompanied by multiple hydrophobic contacts involving Ala17, Val90, Lys91, Tyr89, Tyr90, and Tyr98. Additional van der Waals interactions were observed with Glu70 and Arg68, contributing to overall binding stability within the PB1 pocket (Fig. 8 and Table. S6). The clustering of hydrophobic and aromatic residues around the ligand suggests deep insertion into a non-polar cavity, consistent with a stable hydrophobic core.

Similarly, ZINC08877690 (– 9.1 kcal/mol) establishes a strong π–cation interaction with Arg96, supported by π–σ, alkyl, and π–alkyl interactions involving Ala17, Tyr9, Tyr98, Val90, and Lys91. Additional van der Waals contacts with Glu70, Asp92, and Arg68 further stabilize the complex (Fig. 8 and Table. S6). The combination of aromatic stacking and charged interactions indicates a robust and complementary fit within the PB1 binding pocket.

Collectively, these results identify ZINC70669789 and ZINC08877690 as promising small-molecule inhibitors of p62, demonstrating high binding affinity, favorable interaction profiles, and complementary engagement of both polar and hydrophobic residues critical for PB1-domain stability.

## Discussion

In this study, we performed a comprehensive pan-cancer analysis of *p62* to elucidate its genomic alterations, expression patterns, clinicopathological relevance, and molecular mechanisms using TCGA datasets. Overall, *p62* exhibited a low mutation frequency (0.69%) across cancer types, in contrast to frequently mutated genes (> 10%), highlighting its conserved functional importance. Moreover, gene amplification was rare except in ACC, KIRC, and USC. The high amplification rate in ACC likely reflects the general chromosomal instability characteristic of this tumor type [13]. However, these genetic alterations were not associated with patient survival and had no prognostic value in cancer.

At the transcriptomic level, *p62* was significantly upregulated in LIHC, READ, LUAD, BRCA, KIRP, KICH, and KIRC, with elevated expression correlating with poor prognosis, particularly in BRCA and LUAD, consistent with prior reports [33, 71, 78]. Besides, recent studies have further elucidated that the prognostic impact of *p62* largely depends on its subcellular localization. Accordingly, while cytoplasmic accumulation of p62 predicts a worse prognosis, nuclear accumulation of p62 protein appears to have a negligible influence on patient outcomes [109]. Furthermore, high *p62* expression correlated with advanced clinical stage and T stages, in cancers such as BRCA, LUAD, and KIRP, which underscoring its potential diagnostic value.

To further elucidate the role of *p62* in cancer development and progression, we conducted a correlation analysis between *p62* expression and hallmark pathway activity using ssGSEA scores across a pan-cancer cohort. Overall, *p62* showed broad positive correlations with several oncogenic and metabolic pathways in most cancers, including oxidative phosphorylation, ROS pathway, UPR, MYC targets v2, DNA repair, and mTORC1 signaling. These associations suggest that *p62* acts as a central coordinator of metabolic adaptation and stress tolerance in tumor cells.

Oxidative phosphorylation is a key metabolic pathway that fuels energy production and supports tumor proliferation, invasion, and metastasis. Dysregulation of this pathway promotes drug resistance and suppresses immune cell activity within the TME, facilitating immune evasion [79, 106, 107]. The strong positive correlation between *p62* and oxidative phosphorylation suggests that *p62* may enhance mitochondrial function—potentially by preserving mitochondrial integrity through its established role in mitophagy, the selective autophagic removal of damaged mitochondria [55].

In addition to mitophagy, *p62* regulates mTORC1 and NRF2 signaling—two central pathways in cancer metabolic reprogramming [27]. Consistent with this, we observed positive correlations between *p62* and mTORC1 signaling in 21 cancers, in line with prior reports that *p62* activates mTORC1 via interaction with Raptor and regulation of mTOR ubiquitination [7, 17, 85].

On the other hand, during oxidative phosphorylation, ROS are natural byproducts of mitochondrial respiration. While moderate ROS levels promote cell survival and proliferation, excessive accumulation induces oxidative damage and genomic instability [50]. The observed correlation between *p62* and ROS pathways suggests that *p62* contributes to a pro-oxidative, stress-adapted tumor phenotype. Supporting this, *p62* expression correlated positively with TMB and MSI, consistent with elevated genomic instability.

High ROS levels can also induce DNA damage, stimulating compensatory upregulation of DNA repair pathways [50]. In line with this, *p62* showed positive correlations with DNA repair in most cancers. Although previous studies reported that *p62* depletion enhances DNA repair capacity [28], this discrepancy may reflect differences in gene sets—our analysis used the hallmark DNA repair signature, which excludes key homologous recombination genes such as BRCA1/2 [68]. Thus, enhanced DNA repair pathway activity in tumors with high *p62* expression may represent a compensatory response to ROS-induced genomic instability.

Excessive ROS also suppresses immune cell function, including cytotoxic T cells, macrophages, and dendritic cells; thereby fostering an immunosuppressive TME [46]. Additionally, oxidative stress can upregulate immune checkpoint molecules such as PD-L1, contributing to immune escape [22, 82]. Consistent with this, our pan-cancer analysis revealed that in 14 tumor types—BRCA, CESC, COAD, ESCA, HNSC, KIRC, LIHC, LUAD, LUSC, PAAD, PCPG, PRAD, READ, THCA—high p62 expression was associated with increased tumor purity and decreased infiltration immune cells, specifically, of CD8⁺ T cells, CD4⁺ T cells, and dendritic cells. The concordant reduction across immune subsets suggests that *p62* upregulation promotes an immune-desert or immune-excluded phenotype, characterized by poor effector cell recruitment and enhanced immune evasion.

These computational findings are consistent with prior experimental evidence. For instance, *p62* knockdown in breast cancer enhances infiltration of cytotoxic CD8⁺ T cells and increases pro-inflammatory Th1 cytokines such as *IFN-γ*, *TNF-α*, and *IL-12* [78]. Similar trends observations have been reported in hepatocellular carcinoma [56]. Moreover, in gastric cancer, inhibition of autophagy leads to *p62* accumulation and subsequent upregulation of *PD-L1* via NF-κB activation, promoting immune escape [94]. Likewise, in non-small-cell lung cancer, *IL-17A* enhances ROS production and activates NRF2, resulting in p62 accumulation and a suppressive TME [51].

Our study expands these observations by identifying similar associations between *p62* and reduced immune-cell infiltration in ESCA, HNSC, LUSC, PRAD, THCA, KIRC, PAAD, and PCPG, tumor types in which such relationships have not previously been reported. Furthermore, while earlier studies in colorectal cancer did not clearly define the role of *p62* in anti-tumor immunity, our separate analyses of COAD and READ demonstrate that in both contexts, elevated *p62* expression corresponds to diminished T-cell infiltration, reinforcing its contribution to immune evasion.

Mechanistically, this suppression of immune infiltration may be driven by *p62*’s association with increased expression of immunosuppressive checkpoint molecules such as *B7-H3*, *EBAG9*, *PVR*, *TNFRSF14*, *TGFB1*, and *PD-L1*. Previous studies have demonstrated their pivotal roles in T-cell suppression, immune evasion, and tumor progression, and they have emerged as promising immunotherapy targets either either individually or in combination with *PD-L1*/*PD-1* inhibitors [3, 19, 43, 96, 108]. In this regard, our study highlights that in multiple cancer types, including READ, BRCA, COAD, LIHC, ESCA, PRAD, LUSC, and LUAD, *p62* exhibits strong positive correlations with these inhibitory checkpoint genes.

Among them, *B7-H3* has gained particular attention as a next-generation checkpoint target. Recent advances, such as enoblituzumab, a B7-H3-specific monoclonal antibody, and HS-20093, a small-molecule B7-H3 inhibitor, have demonstrated encouraging efficacy in small-cell lung patients [106, 107]. Furthermore, our data reveal that *p62* also positively correlates with *PD-L1,* a well-established target of ICIs [2, 103], across several tumor types, reinforcing its role in maintaining an immunosuppressive tumor microenvironment and underscoring the therapeutic potential of targeting *p62* as part of combined checkpoint inhibition strategies [2, 103].

In addition, our pan-cancer analysis revealed a negative correlation between *p62* and *PD-1* in four cancers (BRCA, LIHC, PRAD, THCA), and with *CTLA-4* in eight cancers (KIRC, ESCA, READ, COAD, PAAD, BRCA, PRAD, THCA). Given that *PD-1* and *CTLA-4* are predominantly expressed on activated or exhausted T cells, their bulk tumor expression largely reflects T-cell infiltration and activation rather than tumor-intrinsic checkpoint regulation [16, 41]. Accordingly, tumors with high *p62* and low immunoscore would be expected to exhibit reduced *PD1* and *CTLA4* expression, simply due to fewer infiltrating T cells within the tumor microenvironment. Notably, tumor-intrinsic checkpoint molecules such as *PD-L1* can be regulated independently of immune infiltration, often upregulated through oncogenic or stress-response pathways such as p62–NRF2–mTORC1 signaling, even when *PD-1* and *CTLA-4* expression decline in immune-cold contexts [9, 88, 100].

Beyond these, *p62* also correlated positively with UPR and MYC target gene sets across multiple cancers, supporting its broader role in promoting metabolic plasticity and proteostatic stress adaptation. In this context, *p62* participates in the UPR by forming aggresome-like structures that sequester misfolded proteins and maintain proteostasis [62]. Although a direct interaction between *p62* and MYC has not been conclusively shown, prior studies suggest *p62* can indirectly sustain MYC-driven transcriptional programs through its regulation of autophagy and mTOR signaling [26, 38] This association may also extend to cellular metabolism, where *p62* function can intersect with MYC-driven metabolic processes [5, 14].

On the other hand, *p62* exhibited positive correlations with several tumor-suppressive and immune-stimulatory pathways, including p53 signaling, apoptosis, interferon-α/γ responses, complement activation, allograft rejection, inflammatory response, and IL6/JAK/STAT3 signaling, in a distinct cluster of cancers. This pattern suggests that, under certain microenvironmental contexts, *p62* may exert context-dependent or even anti-tumor functions, rather than solely promoting oncogenesis and immune evasion.

Notably, within this group, GBM, LGG, OV, SARC, and TGCT showed a positive correlation between *p62* the immunoscore, accompanied by enrichment of CD4⁺ T cells and/or dendritic cells. To our knowledge, no previous studies have reported this p62-associated immune activation signature in these tumor types, indicating a potentially novel and context-specific role.

Although direct experimental links between *p62* and increased immune infiltration remain limited, the observed association of interferon responses, complement, allograft rejection, and p53 signaling with enhanced anti-tumor immunity is well supported by prior studies [1, 15, 53, 69]. However, since our analysis relied on bulk RNA-seq and immune-cell deconvolution using the TIMER algorithm, these findings should be interpreted with caution, as the associations may reflect context-dependent immune states rather than direct functional effects of *p62*.

Taken together, these results underscore the dualistic nature of *p62* in cancer. While in many epithelial malignancies it supports metabolic reprogramming, immune exclusion, and tumor progression, in specific tumor types such as GBM, LGG, OV, SARC, and TGCT, p62 may contribute to immune stimulation and potentially restrain tumor growth.

Based on these findings, targeting *p62* appears to represent a promising therapeutic approach for cancer treatment, particularly when combined with immunotherapy in epithelial cancers. While ICIs have revolutionized cancer therapy by enabling durable responses in subsets of patients [25, 59], a considerable proportion of patients either fail to respond or eventually develop resistance. Consequently, there is increasing emphasis on rational combination strategies aimed at enhancing therapeutic efficacy and overcoming resistance [57].

For instance, ICIs combined with VEGF-targeted agents such as ramucirumab or regorafenib have demonstrated promising results in advanced gastric cancer [83]. Similarly, pairing ICIs with poly (ADP-ribose) polymerase inhibitors in ovarian cancer exploits complementary mechanisms—enhancing immune activation while impairing DNA damage repair pathways [97]. In melanoma, triplet regimens combining BRAF/MEK inhibitors with ICIs have improved progression-free survival and response durability compared with targeted therapy alone [12]. Additionally, in HER2-positive breast cancer, co-targeting HER2 and immune checkpoints underscores the growing recognition of the interplay between oncogenic signaling and immune modulation [74].

In light of these advances, we aimed to identify novel p62 inhibitors that could complement immunotherapeutic approaches by reprogramming the tumor microenvironment toward immune activation. The PB1 domain of p62 is critical for its role as a signaling adaptor, mediating homo- and hetero-oligomerization and enabling p62 to form higher-order complexes that regulate multiple oncogenic and stress-response pathways [63]. To target this domain, we employed a structure-based virtual screening strategy using natural compounds as candidate inhibitors.

Natural products have long been a cornerstone of anticancer drug discovery, accounting for approximately 25% of newly approved oncology drugs and offering structurally diverse scaffolds with high biological relevance [30, 65]. Recent advances in computational modeling and screening technologies have further revitalized the exploration of natural product libraries, enabling the identification of bioactive compounds with novel mechanisms of action, some of which are currently in clinical trials [10, 29].

Accordingly, we utilized the ZINC Natural Products subset, comprising over 80,000 structurally diverse compounds [34], as the basis for our virtual screening pipeline. Besides, AutoDock Vina was chosen for molecular docking due to its proven accuracy and speed in distinguishing active from inactive ligands [37].

In addition, to refine the selection of final candidates, additional filtering criteria were applied. Since natural products often exhibit polypharmacology—interacting with multiple molecular targets [90]—we first performed in silico target validation to minimize off-target effects. Subsequently, comprehensive drug-likeness and ADMET (Absorption, Distribution, Metabolism, Excretion, and Toxicity) profiling was conducted, a critical step in early drug discovery for prioritizing compounds with favorable pharmacokinetic and safety profiles. Established rules, including those by Lipinski, Ghose, Veber, Egan, and Muegge [18, 21, 54, 63, 92], provide empirical benchmarks for oral bioavailability, permeability, and metabolic stability. Deviations from these guidelines often predict poor absorption, low membrane permeability, excessive rigidity, or rapid metabolic clearance, reducing translational potential [31].

In our analysis, the top candidates ZINC70669789 and ZINC08877690 fully complied with Veber, Egan, and Muegge rules, exhibiting only minor deviations in the Lipinski and Ghose filter, mainly related to acceptable molecular weight and log P variations. However, their TPSA values (< 100 Å^2^) and consensus logP (~ 4.5) are compatible with drug-like permeability and potential oral absorption [76, 92, 95]. Moreover, ADVERPred analysis predicted no hepatotoxicity, cardiotoxicity, or mutagenic potential, indicating a favorable safety profile.

Collectively, these findings identify ZINC70669789 and ZINC08877690 as druggable scaffolds that that bind the p62 PB1 domain with high affinity and meet rigorous physicochemical and pharmacokinetic standards. This dual optimization of target engagement and drug-likeness underscores their promise as starting points for medicinal chemistry optimization and subsequent in vitro and in vivo validation. Notably, these compounds may be particularly beneficial for cancers such as READ, COAD, LIHC, PRAD, BRCA, ESCA, THYM, LUAD, and LUSC, in which p62 expression is strongly associated with immune-cell exclusion and checkpoint upregulation.

Finally, this study has several limitations. First, although TCGA provides a large and well-annotated pan-cancer resource, sample sizes for certain rare tumor types (e.g., ACC, USC) remain limited, which may reduce statistical power for subtype-specific analyses; validation in independent clinical cohorts is therefore essential. Second, bulk RNA-seq data cannot distinguish p62 expression between tumor cells, stroma, and immune infiltrates; single-cell studies are needed to clarify cell-type-specific contributions. Third, while virtual screening identified promising p62 PB1 inhibitors, experimental validation of binding affinity, selectivity, and biological activity remains pending. Finally, associations between p62 expression, subcellular localization, and prognosis are observational; causal mechanistic studies using CRISPR-based targeting or localization mutants are required to establish functional relevance.

## Conclusion

In conclusion, this pan-cancer analysis identifies p62/SQSTM1 as a conserved, clinically relevant regulator linking oncogenic metabolism (oxidative phosphorylation, ROS, mTORC1, UPR/MYC, and DNA-repair programs) to the tumor immune microenvironment. In most epithelial cancers, elevated p62 expression corresponds to increased tumor purity, reduced CD8⁺/CD4⁺ T-cell and dendritic-cell infiltration, and enhanced expression of immunosuppressive checkpoints such as PD-L1 and B7-H3, supporting a role in immune exclusion. Conversely, a distinct cluster of tumors (GBM, LGG, OV, SARC, TGCT) exhibits positive associations between p62, immunoscore, and interferon/inflammatory signaling, indicating context-dependent or potentially anti-tumor functions.

These observations suggest that, in cancers where p62 contributes to an immunosuppressive milieu, its inhibition could potentially enhance the efficacy of immunotherapy. Our structure-based virtual screening identified two natural compounds, ZINC70669789 and ZINC08877690, as promising PB1-domain inhibitors with favorable drug-likeness and safety profiles. Collectively, these findings position p62 as a multifunctional biomarker connecting metabolism and immune regulation, as a tractable therapeutic target for rational combination strategies with immune checkpoint blockade. Further mechanistic and experimental validation is warranted to substantiate these associations.

## Supplementary Information

Additional file1 (JPG 724 KB)

Additional file2 (JPG 3928 KB)

Additional file3 (JPG 9653 KB)

Additional file4 (JPG 4198 KB)

Additional file5 (JPG 412 KB)

Additional file6 (JPG 1922 KB)

Additional file7 (JPG 2457 KB)

Additional file8 (XLSX 11 KB)

Additional file9 (XLSX 87 KB)

Additional file10 (XLSX 16 KB)

Additional file11 (XLSX 11 KB)

Additional file12 (XLSX 15 KB)

Additional file13 (XLSX 16 KB)

Additional file14 (CSV 1 KB)

Additional file15 (DOCX 3224 KB)

## Data Availability

All datasets analyzed in this study are publicly available from recognized repositories. Gene expression, somatic mutation, and clinical data for 32 cancer types were obtained from The Cancer Genome Atlas (TCGA) via the Genomic Data Commons Data Portal (https://portal.gdc.cancer.gov/) and the UCSC Xena Browser (https://xenabrowser.net/datapages/). Copy number variation data were retrieved from cBioPortal (https://www.cbioportal.org/). Protein and mRNA expression data in normal cells and tissues were obtained from the Human Protein Atlas (https://www.proteinatlas.org/), which incorporates data from GTEx and the FANTOM5 project. Clinical proteomic data from the Clinical Proteomic Tumor Analysis Consortium (CPTAC) were accessed via the UALCAN portal (http://ualcan.path.uab.edu/analysis-prot.html). Hallmark pathway gene sets were downloaded from the Molecular Signatures Database v7.4 (MSigDB, http://www.gsea-msigdb.org/gsea/msigdb/index.jsp). Finally, the crystal structure of p62 (PDB ID: 4MJS) was obtained from the RCSB Protein Data Bank (https://www.rcsb.org), and natural compound structures were retrieved from the ZINC database (https://zinc.docking.org/).

## Abbreviations

ACC: Adrenocortical carcinoma
BLCA: Bladder urothelial carcinoma
BRCA: Breast invasive carcinoma
CESC: Cervical squamous cell carcinoma and endocervical adenocarcinoma
CHOL: Cholangiocarcinoma
CNV: Copy number variation
COAD: Colon adenocarcinoma
DLBC: Lymphoid neoplasm diffuse large B-cell lymphoma
EMT: Epithelial-mesenchymal transition
ER: Endoplasmic reticulum
ESCA: Esophageal carcinoma
ESTIMATE: Estimation of STromal and Immune cells in MAlignant Tumor tissues using Expression data
FANTOM5: Function annotation of the mammalian genome
GBM: Glioblastoma multiforme
GTEx: Genotype-tissue expression
HNSC: Head and neck squamous cell carcinoma
HPA: Human protein atlas
ICI: Immune checkpoint inhibitor
KEAP1: Kelch-like ECH-associated protein 1
KICH: Kidney chromophobe
KIR: KEAP1-interacting region
KIRC: Kidney renal clear cell carcinoma
KIRP: Kidney renal papillary cell carcinoma
LGG: Brain lower grade glioma
LIHC: Liver hepatocellular carcinoma
LIR: LC3-interacting region
LUAD: Lung adenocarcinoma
LUSC: Lung squamous cell carcinoma
MESO: Mesothelioma
MSI: Microsatellite instability
mTORC1: Mammalian target of rapamycin complex 1
NRF2: Pathway nuclear factor erythroid 2–related factor 2 pathway
OS: Overall survival
OV: Ovarian serous cystadenocarcinoma
PAAD: Pancreatic adenocarcinoma
PB1: Phox and Bem1p
PCPG: Pheochromocytoma and paraganglioma
PRAD: Prostate adenocarcinoma
READ: Rectum adenocarcinoma
SARC: Sarcoma
SKCM: Skin cutaneous melanoma
STAD: Stomach adenocarcinoma
TCGA: The Cancer Genome Atlas
TGCT: Testicular germ cell tumors
THCA: Thyroid carcinoma
THYM: Thymoma
TMB: Tumor mutation burden
TME: Tumor microenvironment
t-SNE: T-distributed stochastic neighbor embedding
UBA: Ubiquitin-associated
UCEC: Uterine corpus endometrial carcinoma
UCS: Uterine carcinosarcoma
UPR: Unfolded protein response
UVM: Uveal melanoma
