# Supplementary figures and images for: Comprehensive pan-cancer analysis of p62 reveals its contribution to shaping tumor microenvironment and anti-tumor immunity
Source: Discov Oncol. 2025 Nov 25;16:2296. doi: 10.1007/s12672-025-04135-1 (PMC12748421; doi:10.1007/s12672-025-04135-1)

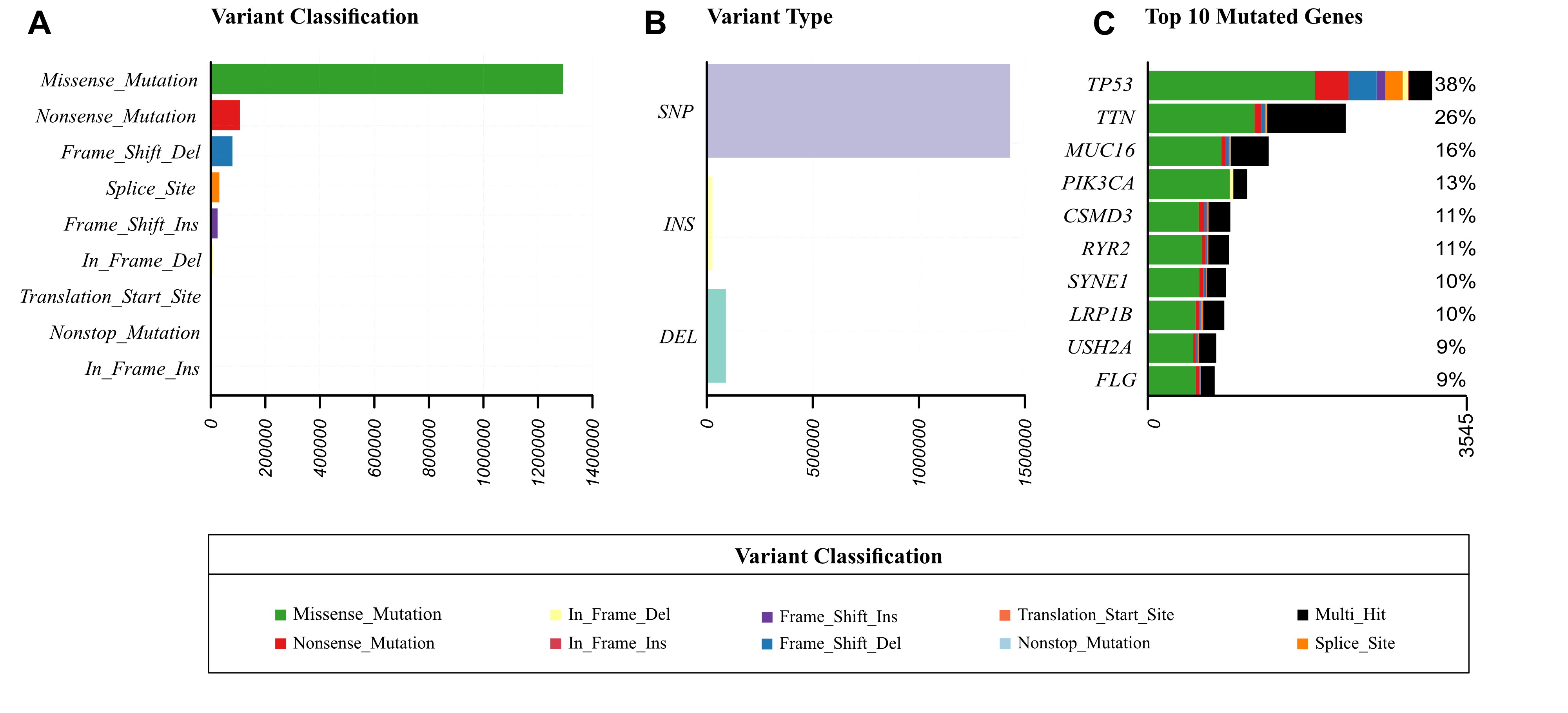

Supplement: Supplementary file 1 — Additional file1 (JPG 724 KB) [file 12672_2025_4135_MOESM1_ESM.jpg]

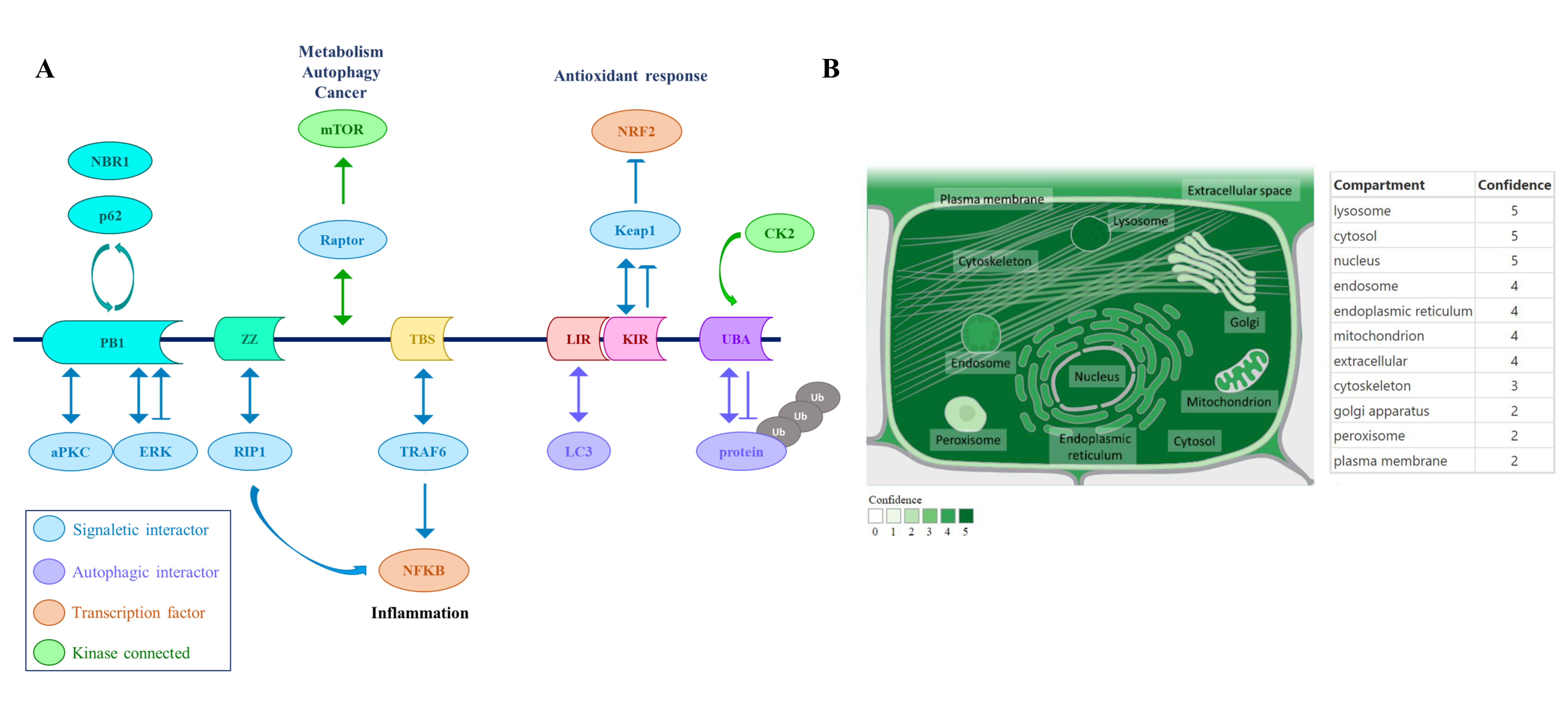

Supplement: Supplementary file 2 — Additional file2 (JPG 3928 KB) [file 12672_2025_4135_MOESM2_ESM.jpg]

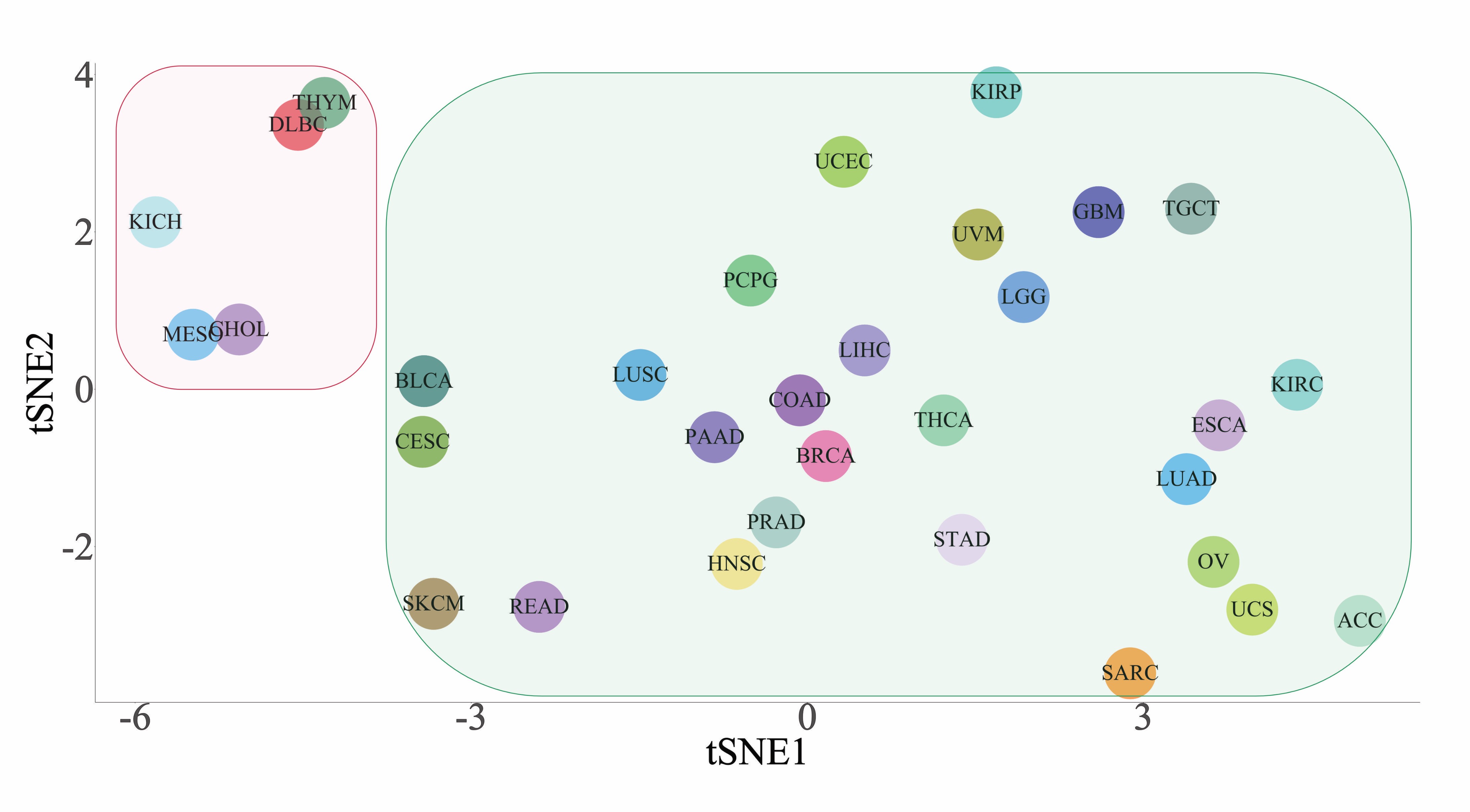

Supplement: Supplementary file 5 — Additional file5 (JPG 412 KB) [file 12672_2025_4135_MOESM5_ESM.jpg]

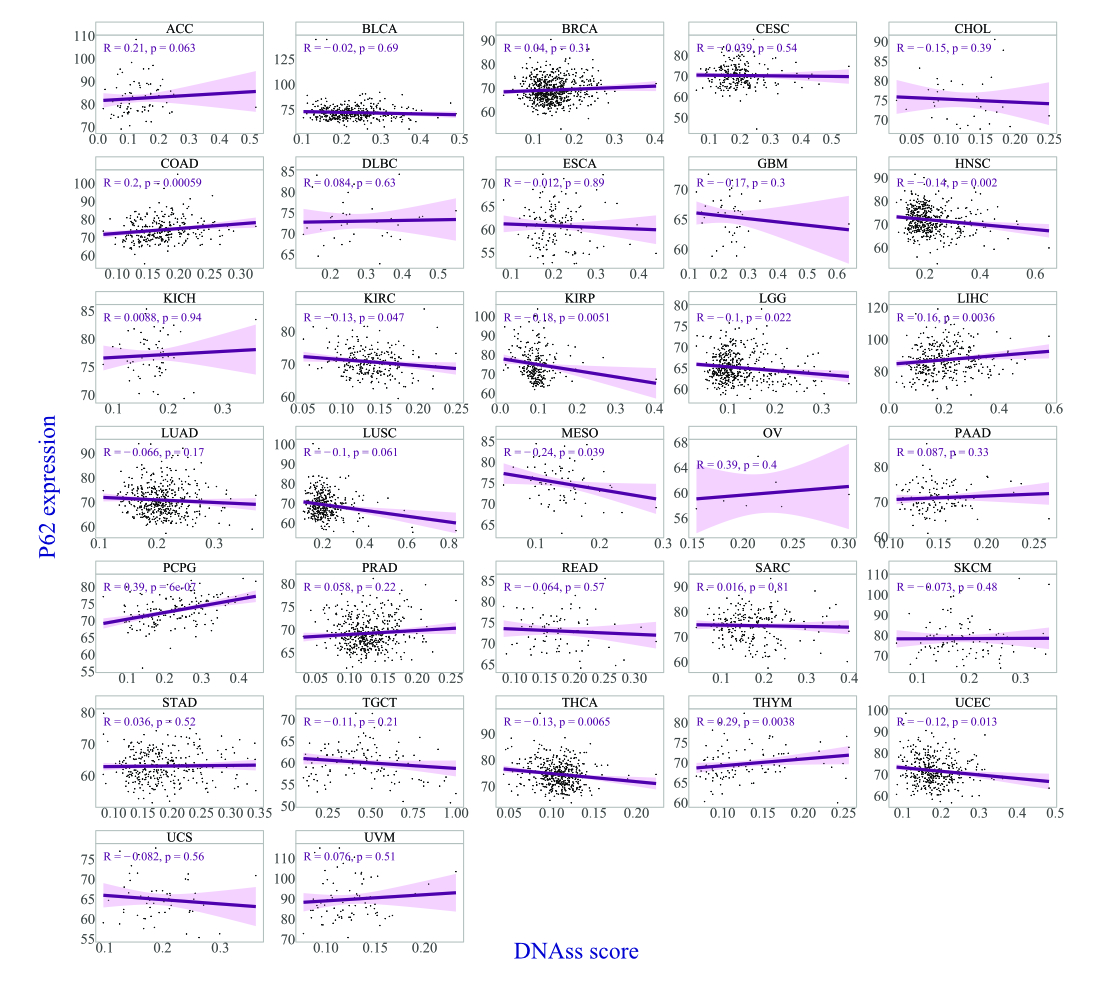

Supplement: Supplementary file 6 — Additional file6 (JPG 1922 KB) [file 12672_2025_4135_MOESM6_ESM.jpg]

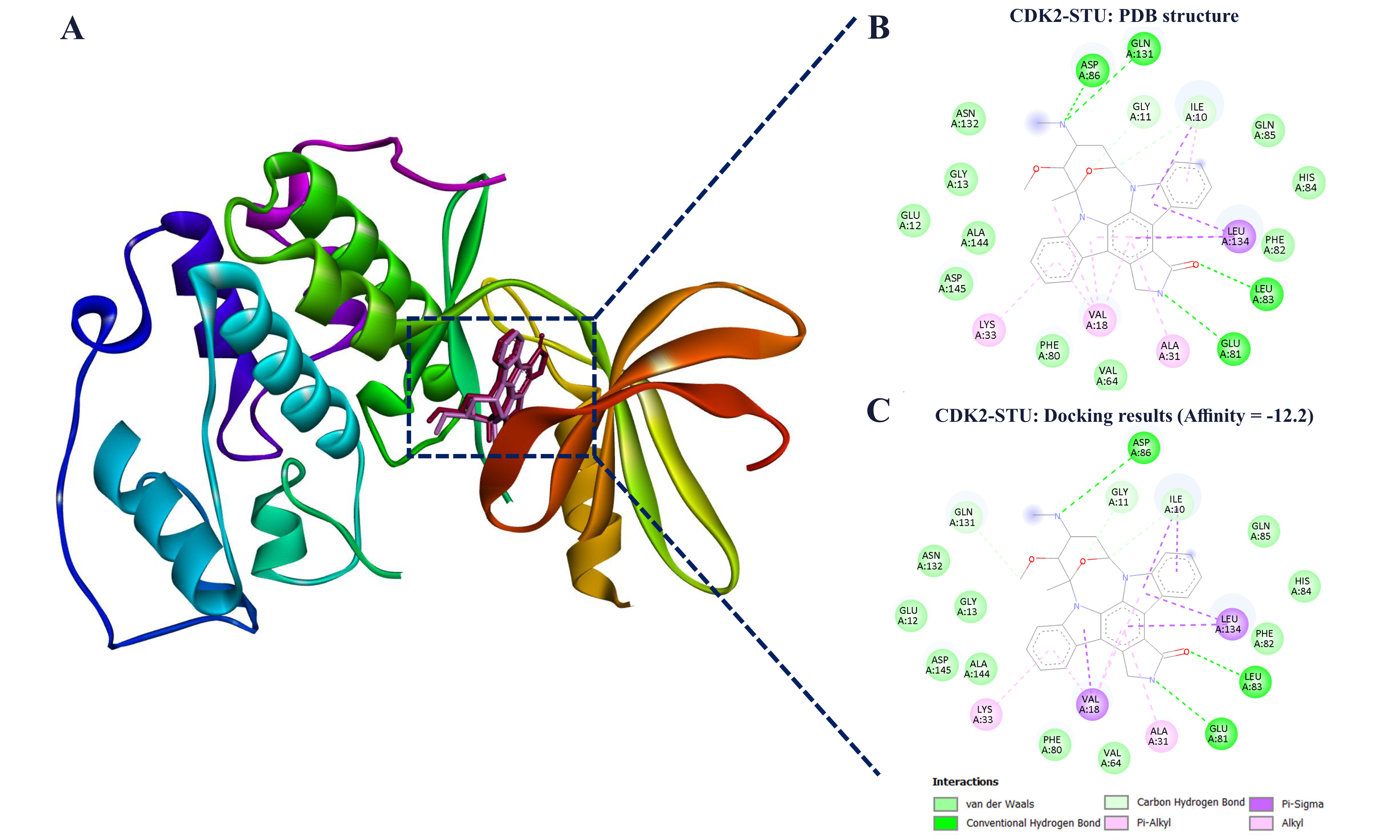

Supplement: Supplementary file 7 — Additional file7 (JPG 2457 KB) [file 12672_2025_4135_MOESM7_ESM.jpg]
