# Supplementary material for: Comprehensive pan-cancer analysis of p62 reveals its contribution to shaping tumor microenvironment and anti-tumor immunity
Source: Discov Oncol. 2025 Nov 25;16:2296. doi: 10.1007/s12672-025-04135-1 (PMC12748421; doi:10.1007/s12672-025-04135-1)
Supplement: Supplementary file 15 — Additional file15 (DOCX 3224 KB) [file 12672_2025_4135_MOESM15_ESM.docx]

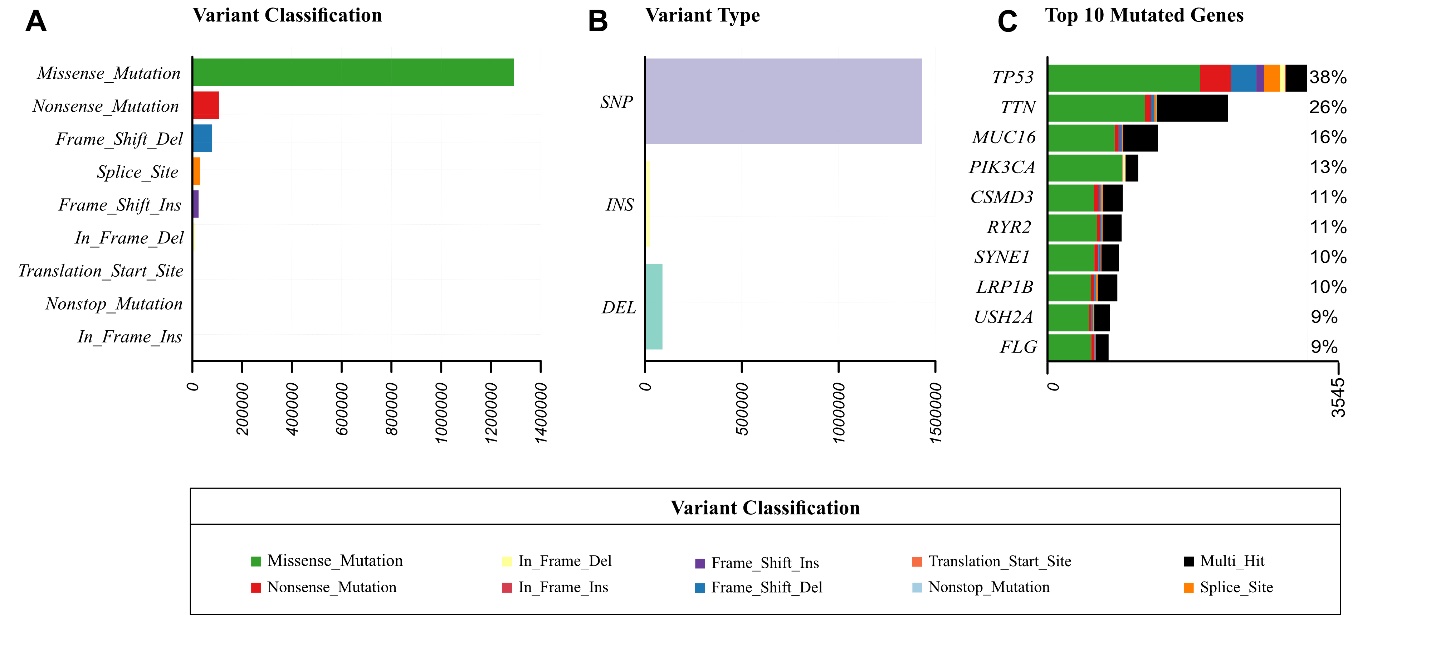


Figure S1. Overview of somatic mutation patterns across TCGA pan-cancer samples. Summary plots display A) the distribution of variant classifications, B) variant types, and C) the top 10 most frequently mutated genes. Missense mutations were the most prevalent variant classification across samples, and single nucleotide polymorphisms (SNPs) were the most common variant type identified. In the mutation summary, different colors represent specific variant classifications, including green (missense), blue (splice site), purple (frameshift), and orange (in-frame mutations). Black bars indicate multi-hit events, in which multiple mutations occur in the same gene within a sample.


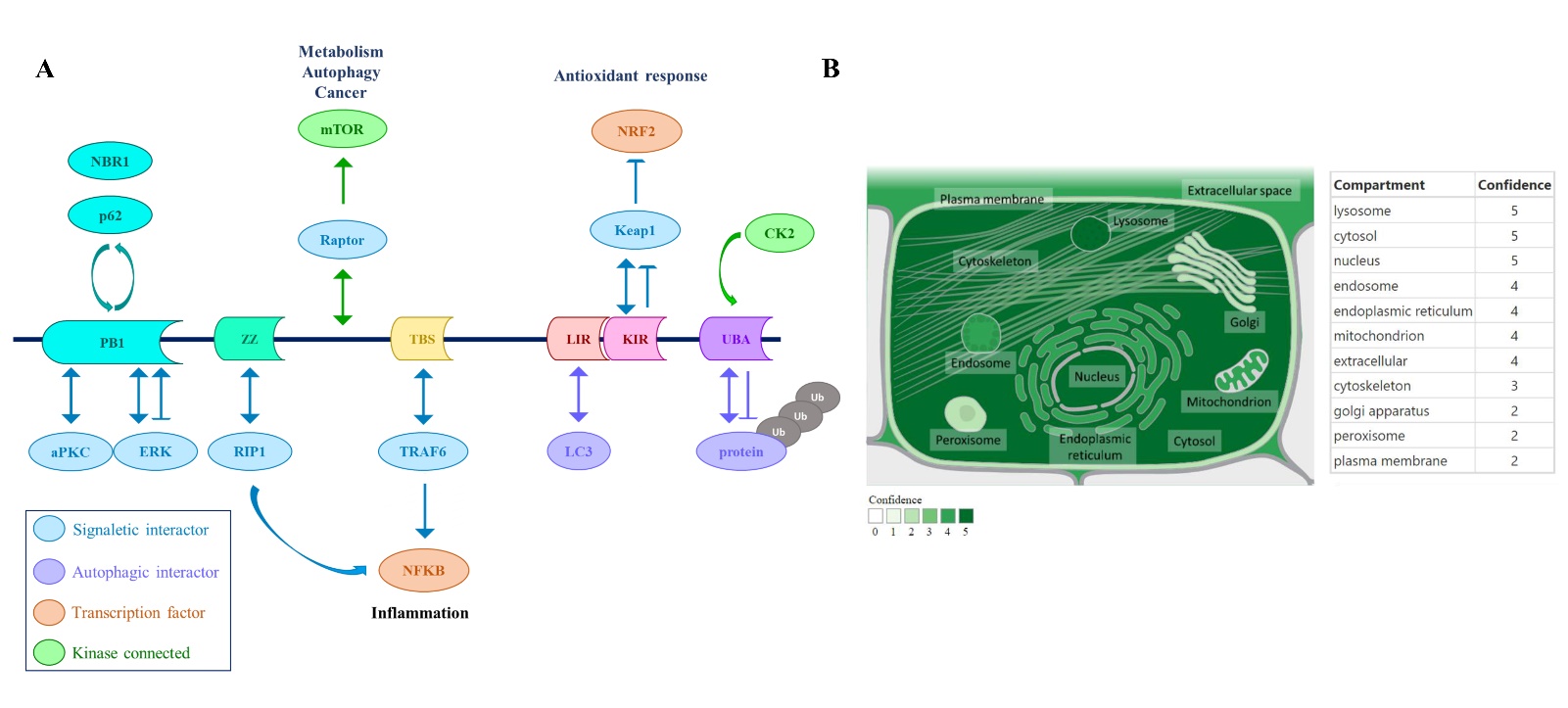


Figure S2. Structural and cellular localization features of p62 protein. A) Schematic representation of p62 protein domains and their known interactors. The protein contains several functional domains, including PB1, ZZ, TBS, LIR, KIR, and UBA, each associated with various signaling, autophagic, and transcriptional pathways. Arrows indicate known interactions, with color-coded categories: signaletic interactors (blue), autophagic interactors (purple), transcription factors (orange), and kinase connections (green). B) Predicted cellular compartment of p62 based on the GeneCards data. Confidence levels (0–5) reflect the prediction strength across multiple compartments, including high-confidence localization in the lysosome, cytosol, and nucleus.


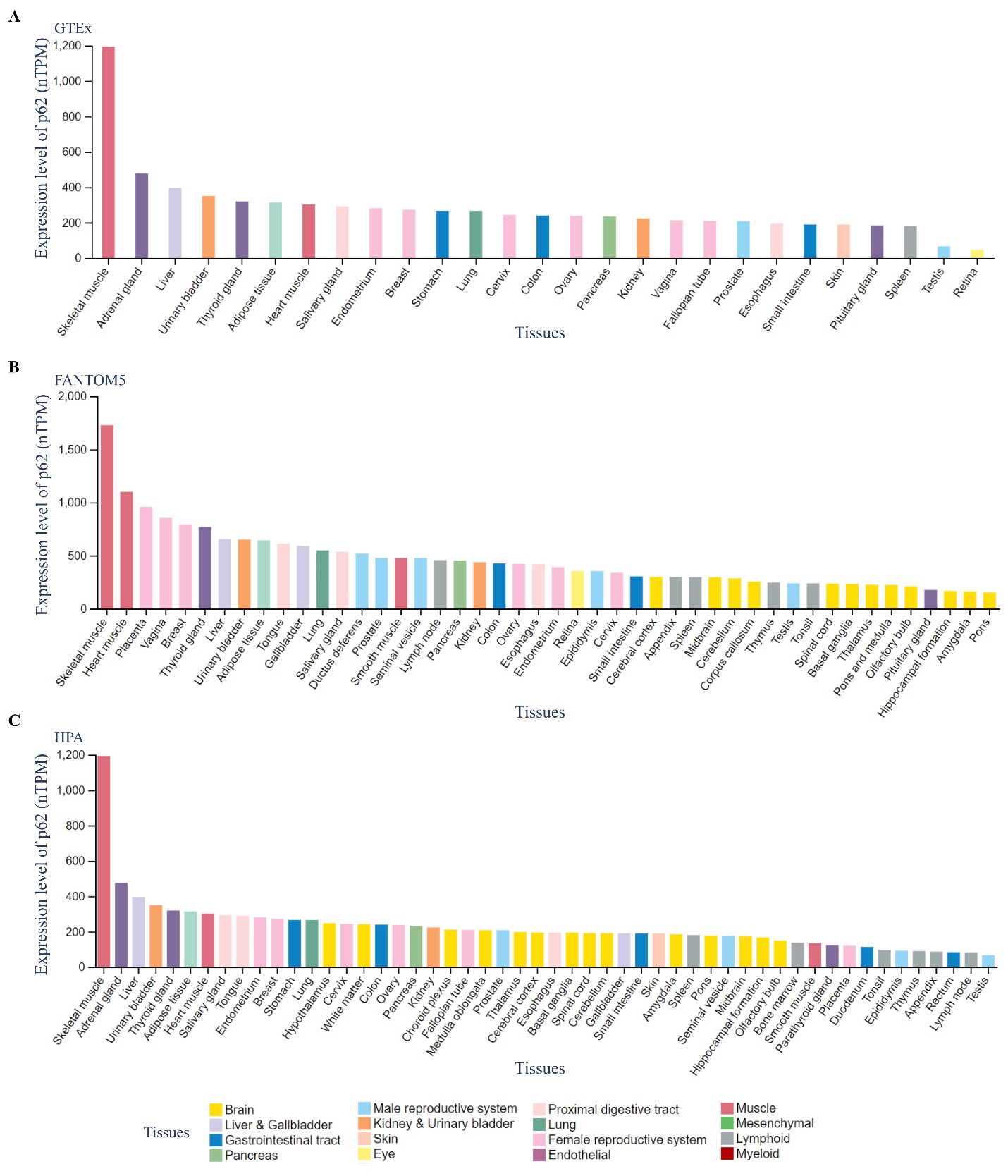


Figure S3. Expression levels of *p62* in normal human tissues based on A) GTEx, B) FANTOM5, and C) Human Protein Atlas (HPA). Across all datasets, p62 exhibited the highest expression in the skeletal muscle.


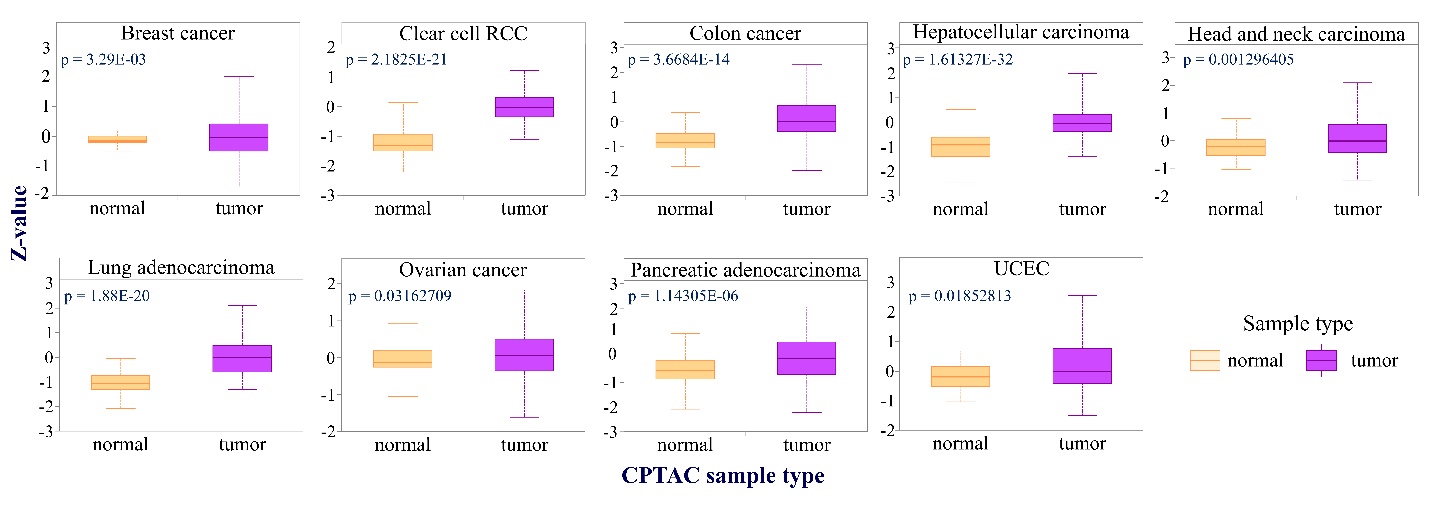


Figure S4. Differential protein expression levels of p62 between tumor and normal tissues across multiple cancer types. Boxplots showing standardized p62 protein expression (Z-values) in tumor versus adjacent normal tissues across various cancer types, based on CPTAC data accessed via the UALCAN web tool. Elevated p62 protein levels were observed in tumor tissues compared to normal tissues in nine of the cancer types analyzed.


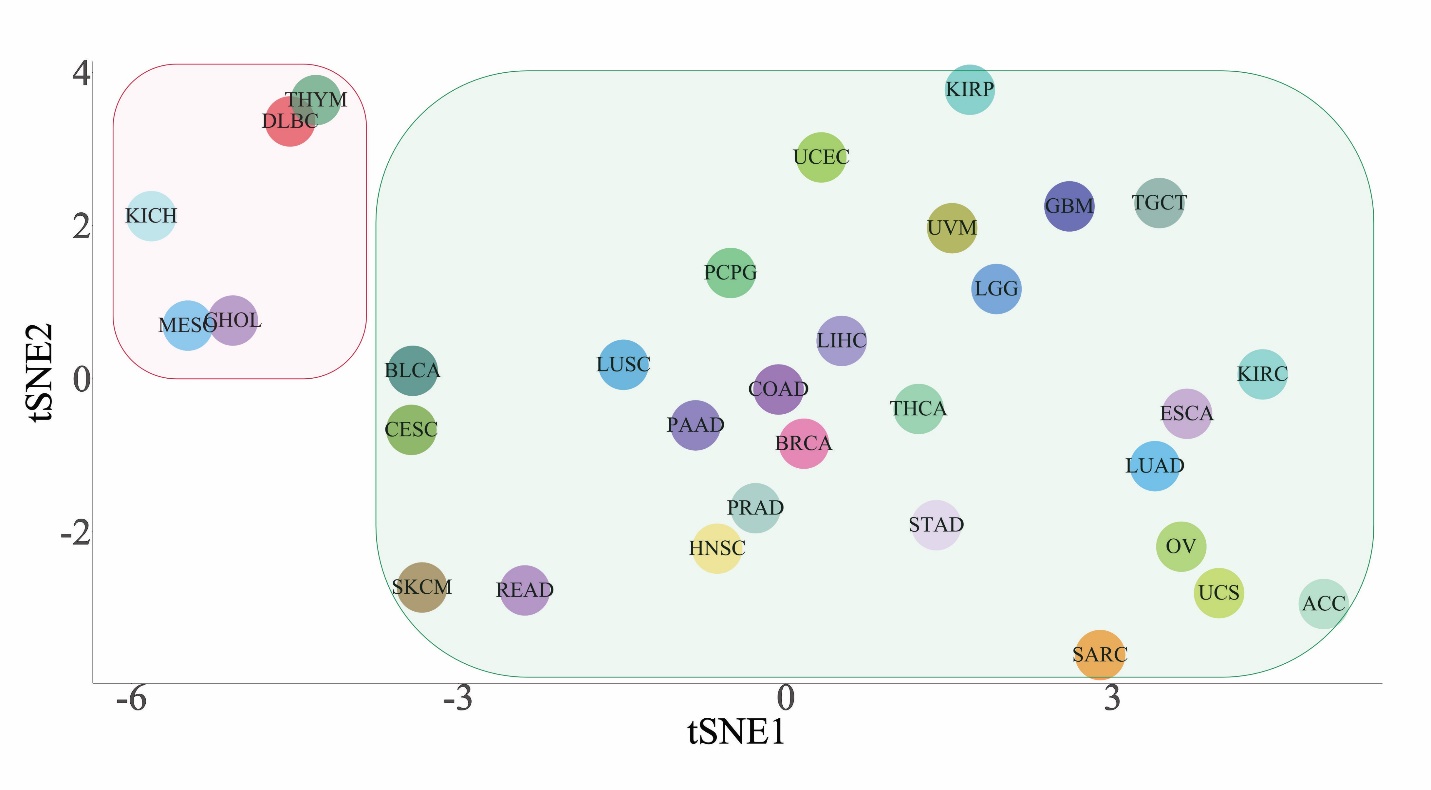


Figure S5. t-SNE plot showing the distribution of cancer types based on p62-associated hallmark pathway enrichment profiles. Each dot represents a cancer type, positioned according to the similarity of the pathway correlation patterns with *p62* expression. Cancers with weak or minimal pathway associations and lower sample sizes, including CHOL, KICH, DLBC, MESO, and THYM, clustered separately and were excluded from downstream clustering analyses (Fig. 4B) to minimize sample size bias.


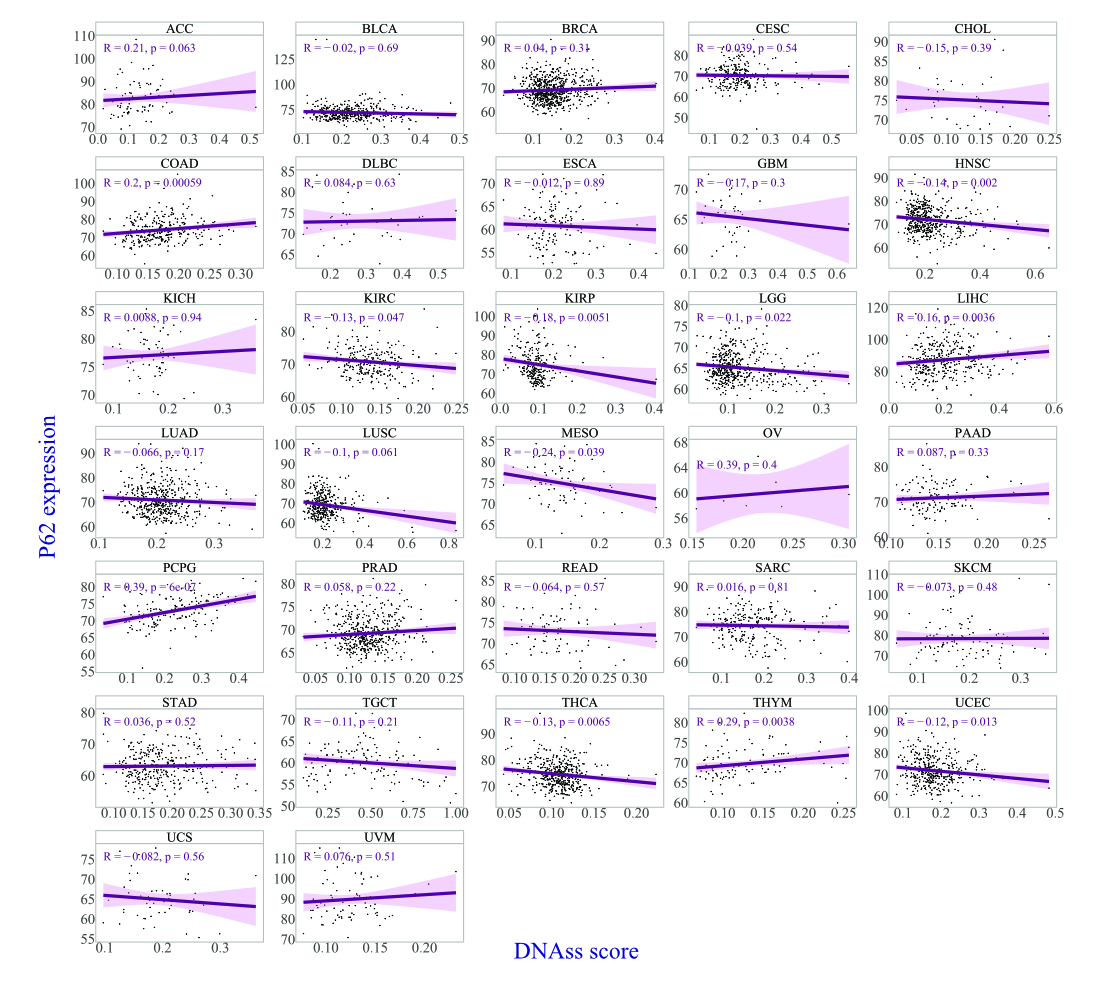


Figure S6. Correlation between p62 expression and cancer stemness scores in different cancer types. Scatter plots showing the Spearman correlation between *p62* expression and stemness scores derived from DNA methylation (DNAss) data.


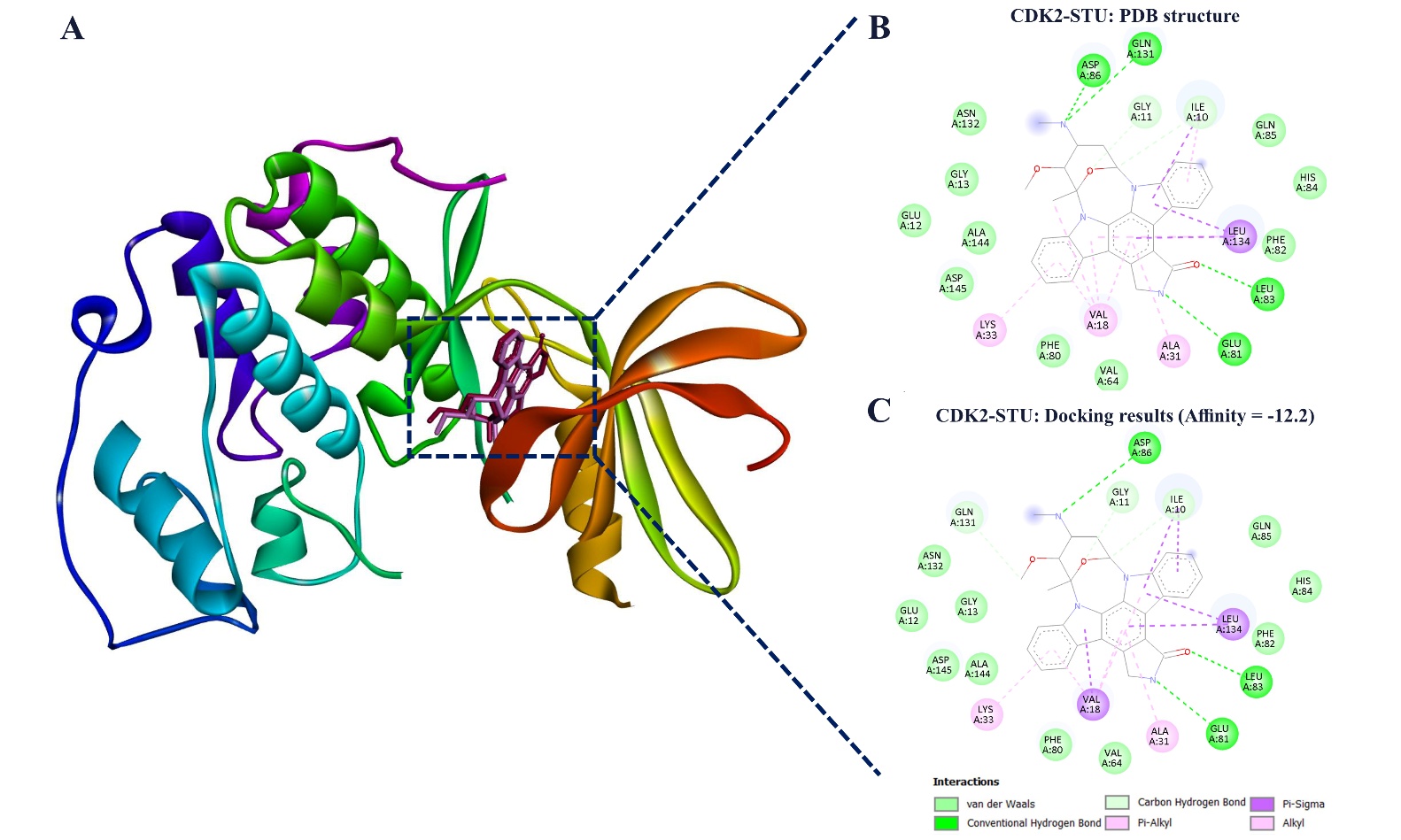


Figure S7. Validation of docking protocol using the CDK2–staurosporine complex (PDB ID: 1AQ1). A) Three-dimensional representation of the CDK2–staurosporine structure, showing the native crystallographic ligand (red) and the redocked ligand obtained from AutoDock Vina (pink). The close overlap between the two confirms accurate reproduction of the experimental binding pose. B) Two-dimensional interaction maps of the native (top) and C) redocked staurosporine–CDK2 complexes, illustrating key interactions. The predicted binding affinity for the redocked complex was –12.2 kcal/mol, demonstrating high fidelity of the docking workflow.
